# Synthesis and bio-properties of 4-piperidone containing compounds as curcumin mimics

**DOI:** 10.1039/d2ra05518j

**Published:** 2022-10-31

**Authors:** Adel S. Girgis, Padraig D'Arcy, Dalia R. Aboshouk, Mohamed S. Bekheit

**Affiliations:** Department of Pesticide Chemistry, National Research Centre Dokki Giza 12622 Egypt girgisas10@yahoo.com as.girgis@nrc.sci.eg; Department of Biomedical and Clinical Sciences, Linköping University SE-581 83 Linköping Sweden

## Abstract

The broad spectrum of curcumin's beneficial properties has encouraged medicinal researchers to investigate its therapeutic efficacy against diverse diseases. The clinical potential of curcumin is, however limited due to its poor pharmacodynamic/pharmacokinetic properties (such as low solubility, pH instability, poor absorption in circulation, rapid elimination from the body and photochemical degradation). 3,5-Bis(ylidene)-4-piperidone scaffolds are considered a curcumin mimic that exhibit diverse bio-properties. The current review provides a brief overview of these mimics and highlights biological activities relevant to drug development.

## Abstract 2

3,5-Diyliden-4-piperidone scaffold are considered as curcumin mimic exhibiting diverse bio-properties.

## Graphical abstract

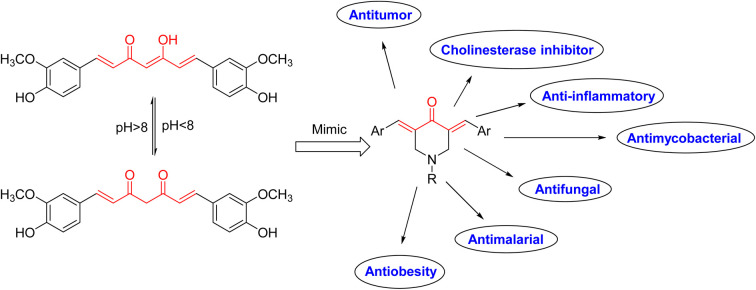

## 1. Introduction

**Fig. 1:**
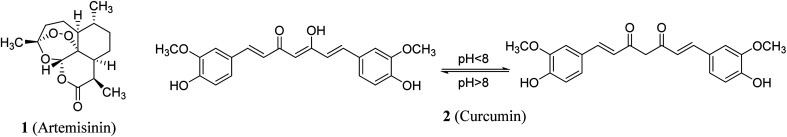
Artemisinin and curcumin, examples of biologically important natural compounds.

Natural products remain one of the main resources for a variety of diverse human needs. Many natural compounds are used directly as drugs or have inspired the development of potent biologically active agents for clinically use. Artemisinin 1 (Fig. 1) is a classic example which was initially extracted from *Artemisia annua* and subsequently approved as an anti-malarial (*Plasmodium falciparum*) drug. The discoverer of artemisinin was granted the Nobel Prize in 2015 in recognition of contribution to medicine.^1^ Curcumin 2 is another famous example of a natural compound with clinical potential. Curcumin was extracted from roots/rhizomes of *Curcuma longa* and has earned a high reputation among medicinal chemists due to its usage in Ayurvedic medicine, as a food additive and as a dye in many Asian countries. Historically, it was first described over 4000 years ago in ancient India where its usage was associated with religious practice.^2^

The broad spectrum of curcumin's beneficial properties has encouraged medicinal researchers into its therapeutic efficacy against diverse diseases. It has been reported to possess anti-inflammatory properties and its sodium salt derivative modulates iNOS and COX-2 (cyclooxygenase-2) gene expression in cultured RAW 264.7 cells.^3^ Antioxidant properties determined by the DPPH (1,1-diphenyl-2-picryl-hydrazyl), ABTS [2,2-azino-bis(3-ethylbenzthiazoline-6-sulfonic acid)], ROO˙ (TRAP) and O^2^˙ (NET) assays have also shown a free radical scavenging effect of curcumin.^4^ Antiproliferative properties of curcumin against many human cancer cell lines [SMMC-7721 (hepatoma), MCF-7 (breast), PC-3 (prostate), NCI-H460 (non-small cell lung) and K562 (chronic myeloid leukemia)] have been reported.^4,5^ In addition curcumin has been shown to display anti-malarial properties against *Plasmodium falciparum*.^6^

Although an enormous amount of research effort has been invested in bringing curcumin and its analogs towards clinical use, thus far no curcumin based product has been approved for use. Over 120 clinical trials on curcumin have failed and consumed federal funds equivalent to 150 million dollars as stated in NIH reports from the last 25 years. This is in part attributed to the promising *in vitro* results obtained in pre-clinical studies, but poor *in vivo* activities. Thus far curcumin has only been approved as a dietary supplement.^1^

The next section highlights some of the important findings of the biological properties of curcumin reported within the last decades with focus on the promising bio-properties of curcumin and its analogs and the potential in identifying more effective hits/leads.

## 2. Curcumin bio-availability

It has been reported that the major clinical limitation of curcumin is due to poor pharmacological properties such as poor pharmacodynamics/pharmacokinetics, low solubility,^7^ pH instability,^8^ poor absorption in circulation, rapid elimination^9^ and photochemical degradation (giving rise to vanillin, ferulic acid, and other small phenols).^10^

The bioavailability of curcumin has been intensively studied. In a phase I clinical trial, Sharma *et al.* reported that the production of the prostaglandin E_2_ (PGE_2_) was (57–62%) decreased in blood samples after 1 h oral administration in patients (with colorectal cancer). Mild diarrhea and discernible toxicity was detected at doses of 0.5–3.6 g daily administration up to four months. Curcumin and its conjugates were detected in plasma and urine and also noticed in patient feces. The entire study concluded that doses of 3.6 g curcumin are recommended for more systemic pharmacological antitumor studies. Additionally, low oral bioavailability (in both animal and human) is supported probably due to intestinal metabolism. The observed bio-properties of curcumin (anti-inflammatory and anticancer) can be attributed to its antioxidant capacity in neutral and acidic pH.^11–13^

Another study investigated the bioavailability of curcumin in rats (*in vivo*) utilizing high performance liquid chromatography. Disappearance of curcumin was noticed from the rat's plasma within one hour of dosing (*i.v.* 40 mg kg^−1^). However, it has been detected in plasma upon p.o. 400 mg kg^−1^ administration suggesting that the gastrointestinal tract is more exposed to the unmetabolized curcumin compared to other tissues.^14^

**Fig. 2:**
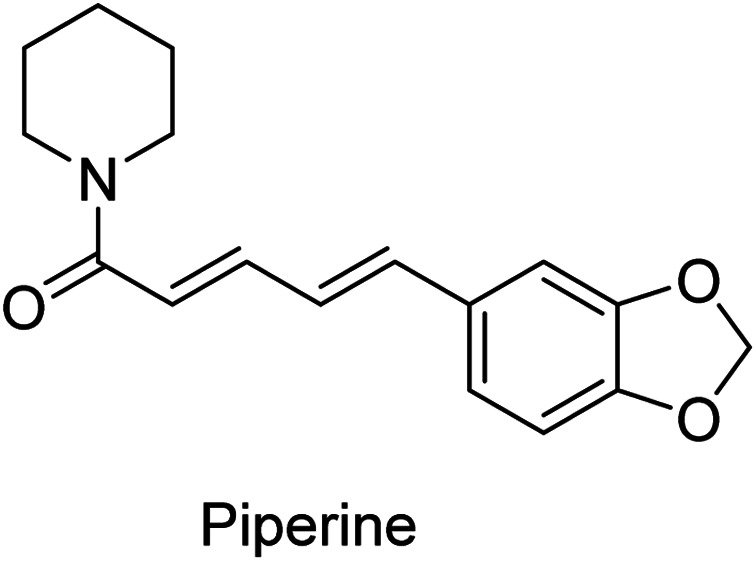
Piperine, a natural compound useable in traditional medicine, responsible for the pungency of black and long pepper.

Low solubility is one of the major limiting parameters for curcumin's therapeutic use. Different approaches have been considered to overcome this problem and improve bioavailability. Piperine (Fig. 2) has been considered for concomitant administration with curcumin, due to the inhibitory properties in both hepatic and intestinal glucuronidation. Increased serum curcumin was noted upon oral administration (2 g kg^−1^) with piperine (20 mg kg^−1^) in rats, with a significant reduction in the half time of clearance. Similar observations were noticed in human volunteers with slight differences. Time to reach curcumin maximum serum concentration was shorter (earlier) in humans than rats, presumably due to physiological differences. Also, the elimination (clearance) time was shorter in humans than rats.^15^

**Fig. 3:**
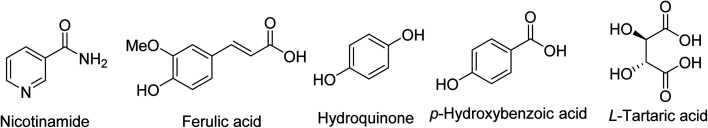
Agents considered for mechano-chemical grinding curcumin co-crystals.

Another approach considered the mechano-chemical grinding of curcumin with diverse agents (nicotinamide, ferulic acid, hydroquinone, *p*-hydroxybenzoic acid and l-tartaric acid) (Fig. 3) in different stoichiometry ratios in an attempt to optimize the physico-chemical properties accessible for solid state oral dosage application. Significant enhancement in solubility and dissolution rates were noticed of the binary eutectics co-crystalline solids.^16^

Another study mentioned the possibility of water solubility enhancement through nano-vehicles with curcumin encapsulated in liposomes, exosomes, dendrimers and micelles.^17^ Curcumin encapsulation in liposomes modified with DDAB (didecyldimethylammonium bromide) was studied using cervical cancer cell lines (HeLa, SiHa). It was observed that the uptake of DDAB liposomes was better than the non-modified ones but more toxic. Additionally, curcumin was released at a faster rate from cationic DDAB liposomes presumably due to the decrease in interaction of the lipid chains as a result of cationic charges.^18^

**Fig. 4:**
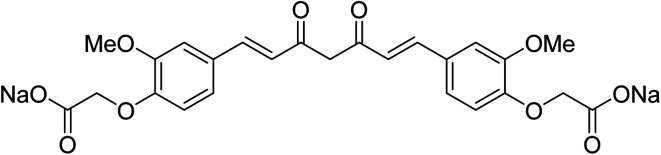
Sodium salt curcumin diacetate.

Sodium salt curcumin diacetate (Fig. 4) was also mentioned as an improved water soluble bio-active agent. Enhancement was noticed relative to that of curcumin itself in aqueous conditions and ability to protect lipid membranes. However, further detailed studies were recommended to support the accessibility for application.^19^ Other studies also adopted bioavailability enhancement of curcumin *via* combination with cyclodextrin,^20^ conjugation with biopolymers^21^ or composite nanoparticles.^22^

## 3. Curcumin chemical structure modification

**Fig. 5:**
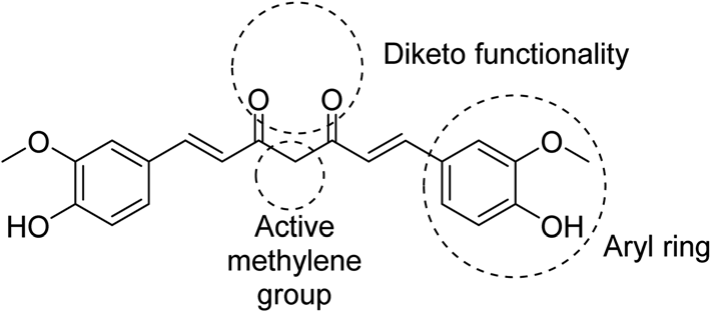
Structure of curcumin indicating the major active sites.

Many efforts have been directed towards designing novel bio-active agents of enhanced potency and better bioavailability to overcome the drawbacks of curcumin. Manipulation of curcumin chemical structure is usually focused on the aryl rings, carbonyl groups, active methylene or the carbon linker (Fig. 5). Most of the approaches of curcumin chemical structure alterations can be summarized in one of the following approaches.^9^

- Modification of the main curcumin skeletal entities.

- Conjugation with other moieties.

- Curcumin mimics.

## 4. Curcumin derivatives with potential biological properties

**Scheme 1:**
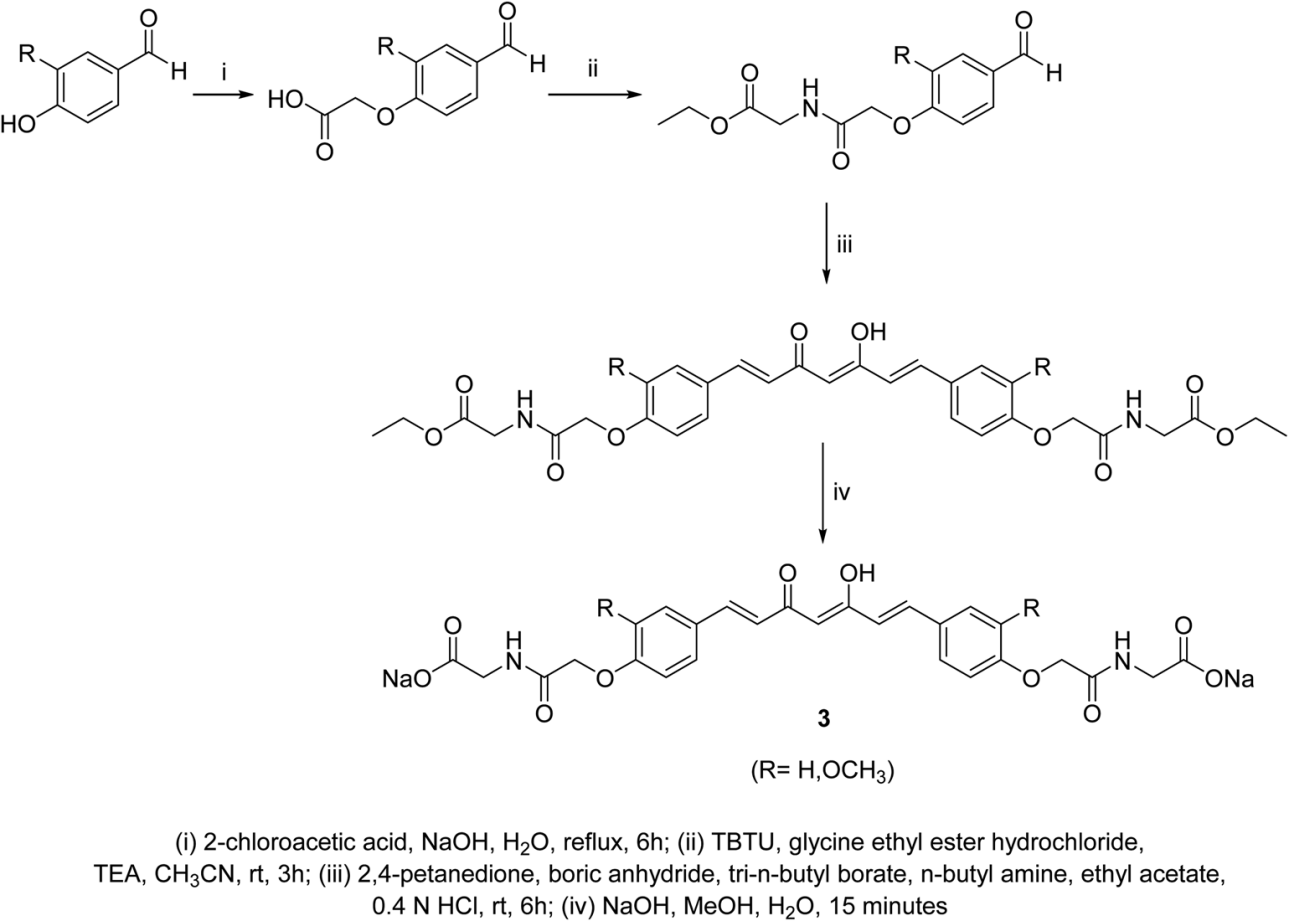
Synthetic pathway of water-soluble curcumin connected to amino acid sodium salts 3.

Curcumin connected to amino acid sodium salts 3 were reported as water-soluble agents. Scheme 1 depicted the synthetic pathway *via* alkylation of the appropriate aldehyde with chloroacetic acid in the presence of NaOH followed by reaction with glycine ethyl ester hydrochloride. Reaction with 2,4-pentandione followed by hydrolysis with methanolic NaOH afforded the targeted agents 3. The synthesized water-soluble agents displayed enhanced antiproliferative properties (MTT assay) against HeLa (cervical cancer) cells (IC_50_ = 0.5 μM for both the synthesized agents) relative to curcumin (IC_50_ = 4.33 μM) with induction of p53 activity, p21 expression and mediated apoptosis. The p53 is the tumor suppressor protein capable for induction of genes controlling cell cycle and apoptosis. It usually arrests the cell cycle at G2/M phase/transition affecting cyclin dependent kinase (Cdc2) necessary for mitosis.^23^

**Scheme 2:**
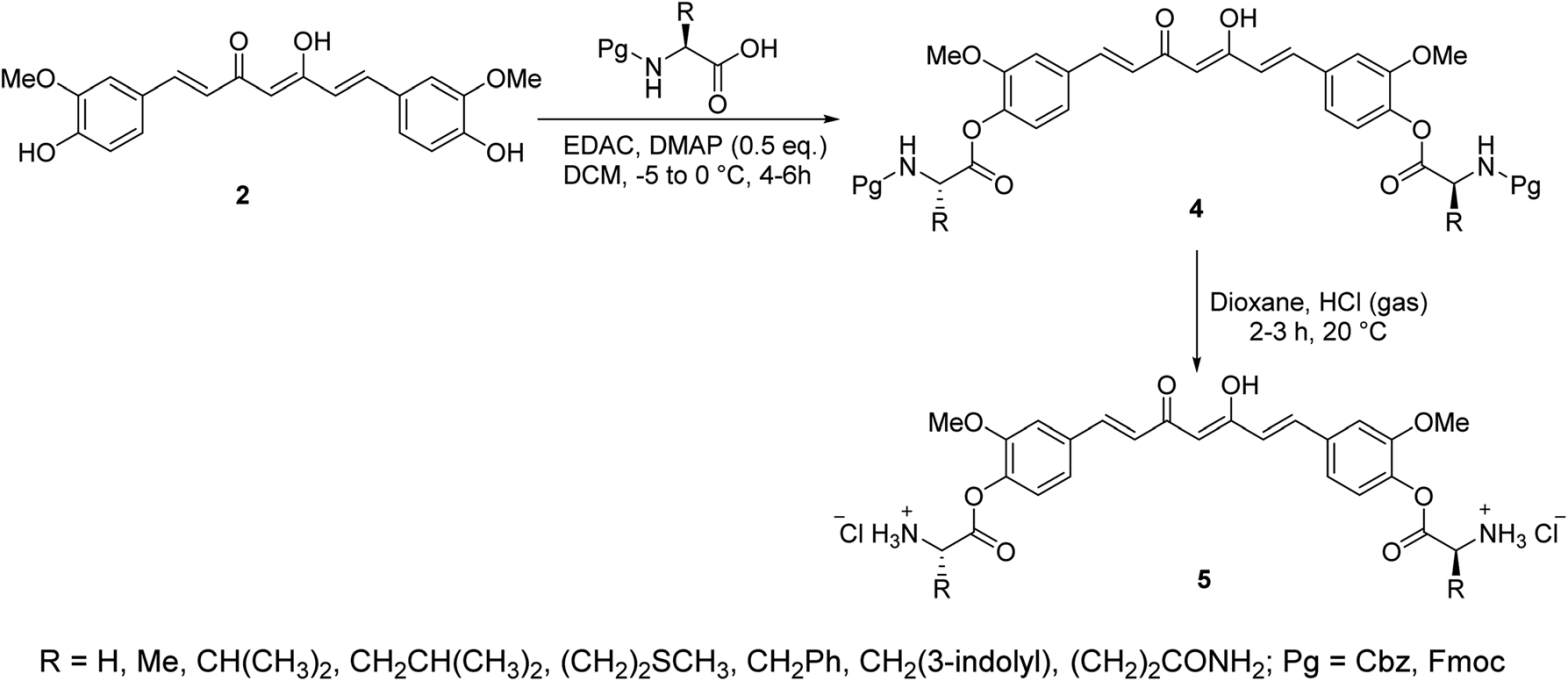
Synthetic route towards curcumin-amino acid conjugates 4 and 5.

A series of curcumin–amino acid conjugates 4 were synthesized through reaction of curcumin 2 with the corresponding protected amino acid (benzyloxycarbonyl “Cbz” and fluorenylmethyloxycarbonyl “Fmoc”) in the presence of EDAC [1-ethyl-3-(3-dimethylaminopropyl)carbodiimide] and DMAP (4-dimethylaminopyridine) at −5 to 0 °C. The unprotected curcumin-amino acid conjugates 5 were obtained from the Boc (*ter*-butyloxycarbonyl) protected analogs (Scheme 2). Some of the synthesized conjugates revealed anti-inflammatory properties (acute carrageenan-induced paw edema in rats) with potency higher than curcumin itself and the standard references used (indomethacin and ibuprofen, clinically used non-steroidal anti-inflammatory drugs). These agents also displayed minor or no ulcerations or lesions on the gastric mucosa of the animals tested, supporting the enhanced bio-properties of the synthesized agents. Additionally, enhanced peripheral (acetic acid-induced abdominal writhing methodology) and central (hot plate technique) analgesic properties were also revealed by some of the synthesized conjugates comparable to curcumin, indomethacin and ibuprofen. The anti-inflammatory properties observed were correlated with the nitric oxide production by lipopolysaccharide-stimulated peritoneal macrophages. Most of the synthesized conjugates showed high antibacterial properties against *S. aureus*, *S. pyogenes* (Gram-positive) and *S. typhi*, *P. aeruginosa* (Gram-negative) bacteria with potency higher than norfloxacin and ciprofloxacin (standard reference, antibiotic useable drugs).^24^

**Fig. 6:**
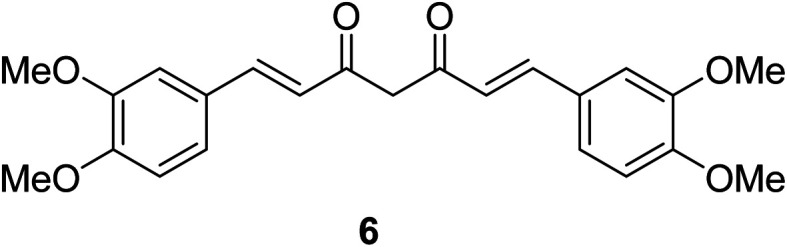
Dimethoxycurcumin 6.

Dimethoxycurcumin 6 (Fig. 6) revealed antiproliferative properties with efficacy comparable to that of curcumin 2 capable to arrest the cell cycle at S-phase. Dimethoxycurcumin is about three times more metabolically stable relative to curcumin in mice. The mode of action was mentioned through oxidative stress and mitochondrial dysfunction.^25^

**Scheme 3:**
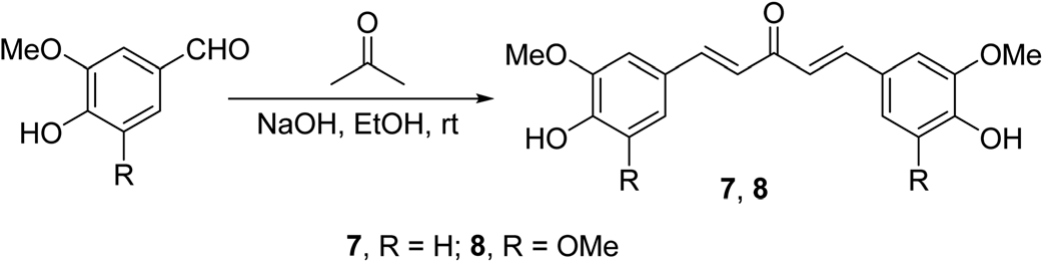
Curcumin analogs 7 and 8.

Curcumin analogs 7 and 8 synthesized through base-catalyzed (NaOH, EtOH) condensation of the appropriate aldehyde with acetone were found to be potent inhibitors (MTT assay) on a variety of human pancreatic cancers (PANC-1, BXPC-3, MIA-PACA-2, ASPC-1, HPAC and HPDE) and apoptosis inducers relative to curcumin^26^ (Scheme 3).

**Scheme 4:**
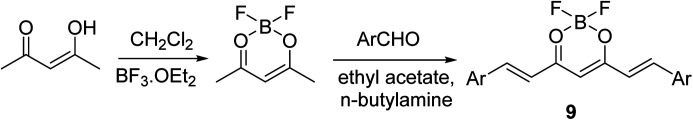
Synthetic route towards the curcuminoid-difluroborons 9.

Curcuminoid-difluoroborons 9 were synthesized through the reaction of acetyl acetone–BF_2_ complex with the appropriate aldehyde under nitrogen atmosphere in ethyl acetate in presence of *n*-butylamine (Scheme 4). Some of the synthesized agents [Ar = 2,3,4-(H_3_CO)_3_C_6_H_2_; 3,4,5-(H_3_CO)_3_C_6_H_2_; 3,4-(H_3_CO)_2_C_6_H_3_; 3-(H_3_CO)-4-(H_3_COO)C_6_H_3_] showed notable antiproliferative properties through NCI (National Cancer Institute) screening program (NCI-60) against a variety of human cancer cell lines (SRB technique).^27^

**Scheme 5:**
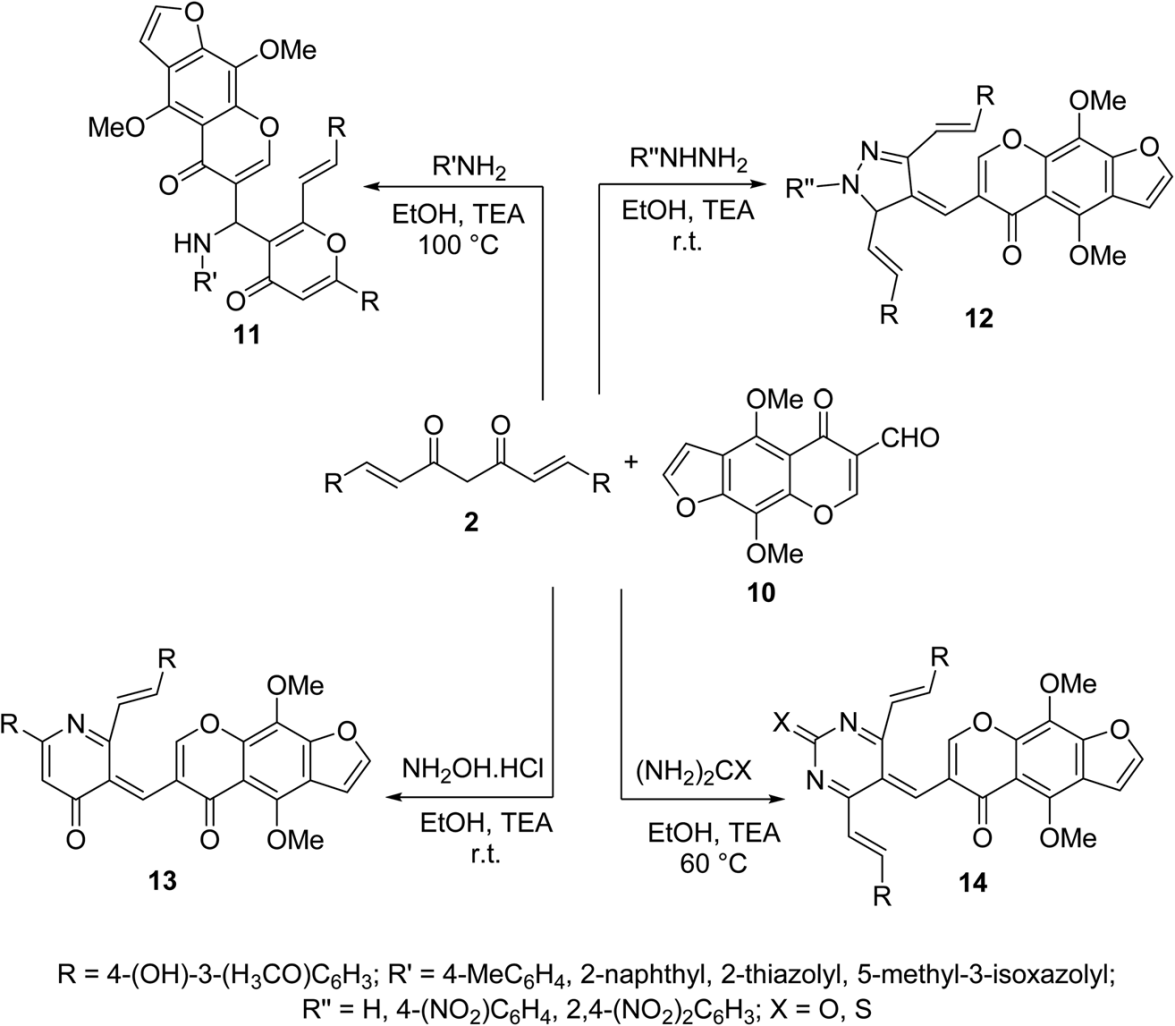
Synthetic route towards furochromone-containing heterocycles 11–14.

Multi-component Biginelli reaction of curcumin with furochromone carbaldehyde 10 and amines, hydrazines, hydroxylamine hydrochloride, urea or thiourea afforded the corresponding furochromone-containing heterocycles 11–14 (Scheme 5). All the synthesized agents showed mild to moderate antiproliferative properties (MTT assay) against MCF7 (breast) and HepG2 (hepatocellular) cancer cell lines relative to doxorubicin and 5-fluorouracil (antitumor standard reference drugs).^28^

**Scheme 6:**
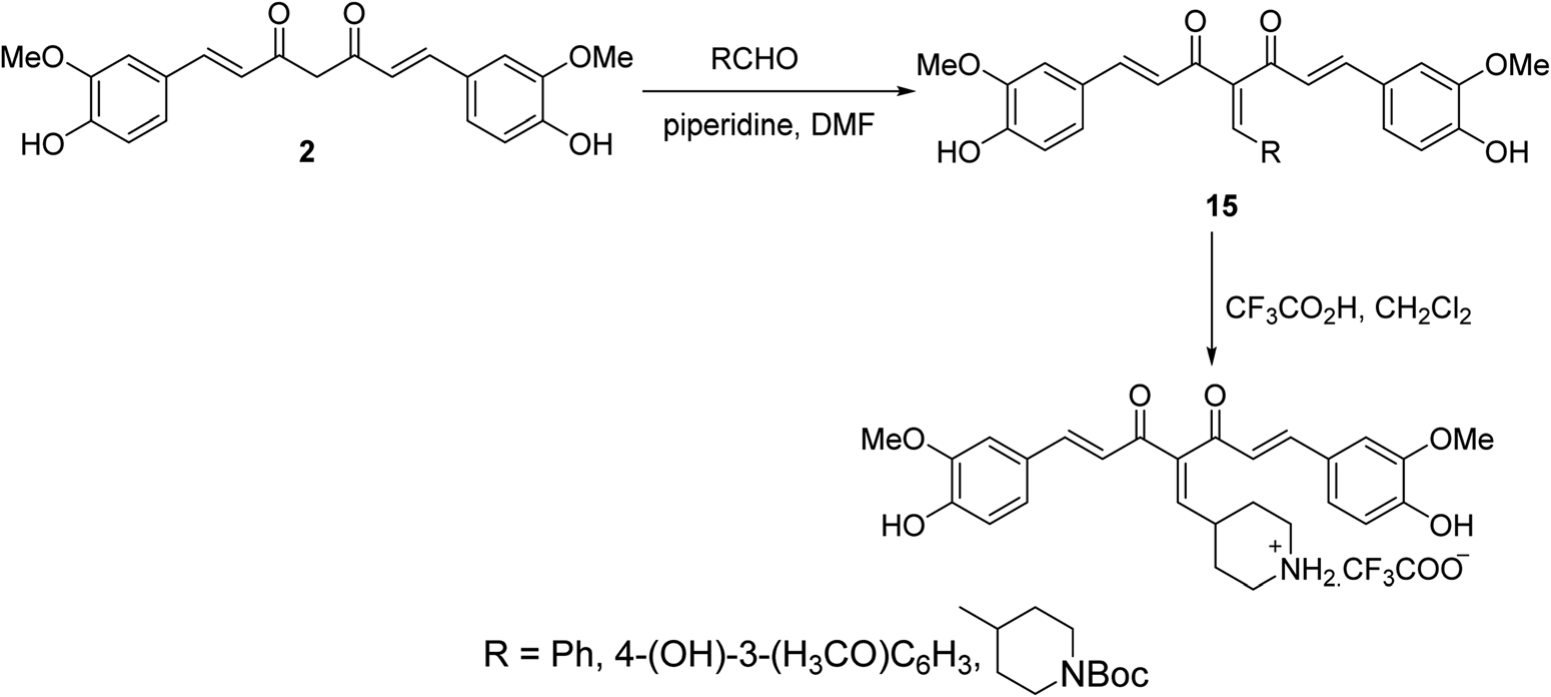
Synthetic route towards curcumin condensates 15.

Knoevenagel condensation of curcumin with the appropriate aldehyde in presence of catalytic amounts of piperidine in DMF afforded the corresponding condensate analogs 15. Trifluoroacetic acid in CH_2_Cl_2_ was used for Boc group removal (Scheme 6). Higher antiproliferation properties were observed by the synthesized agents against MCF7 (breast) cancer cell line (SRB assay) than curcumin itself. It has also been noted that the synthesized condensates affect microtubules and polymerization of purified tubulin in addition to the ability to induce p53 mediated apoptosis in tested cancer cells.^29,30^

**Fig. 7:**
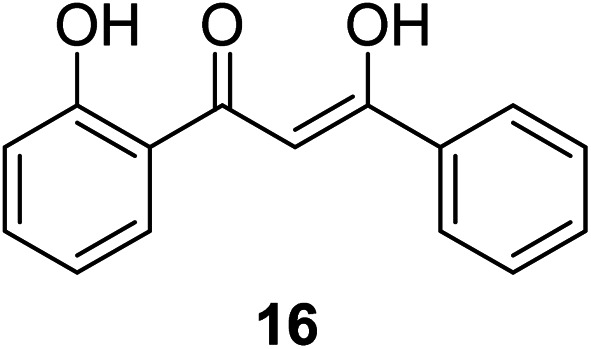
(*Z*)-3-Hydroxyl-1-(2-hydroxyphenyl)-3-phenylprop-2-ene-1-one.

The antiproliferation properties of (*Z*)-3-hydroxy-1-(2-hydroxyphenyl)-3-phenylprop-2-ene-1-one 16 (mimicking curcumin) (Fig. 7) was observed by MTT assay against human colon adenocarcinoma (HT29, SW620) cell lines with potency higher than curcumin. Induction of apoptosis indicated by DNA fragmentation and arresting cell cycle at G0/G1 phase was also observed.^31^

**Fig. 8:**
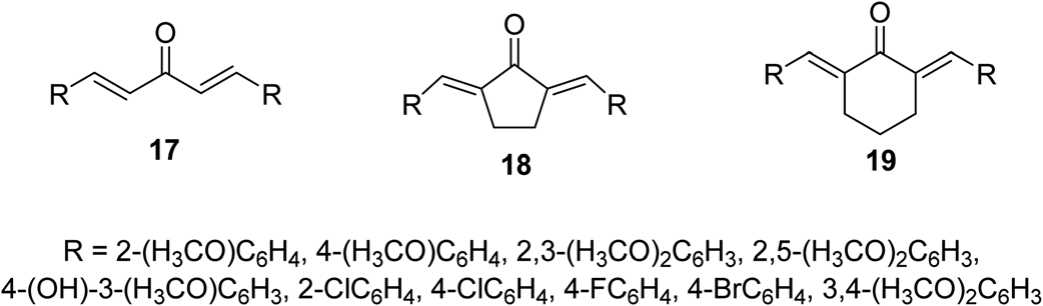
Bis(arylidene)monocarbonyl compounds 17–19 as modified curcuminoid analogs.

Bis(arylidene)monocarbonyl compounds 17–19 were synthesized as modified curcuminoid analogs comprising one ketonic group conjugated with two olefinic groups (Fig. 8) through acid or base catalyzed Claisen Schmidt condensation of the appropriate aldehyde with acetone, cyclopentanone or cyclohexanone. Many of the synthesized agents showed promising antiproliferative properties against MCF7 (estrogen-dependent breast), MDA-MB-231 (estrogen-independent breast), K562 (chronic myelogenous leukemia) and HeLa (cervical) human cancer cell lines (MTT assay) higher than that of curcumin. Some of them revealed higher potency than that of doxorubicin. (1*E*,4*E*)-1,5-Bis(2,5-dimethoxyphenyl)-penta-1,4-dien-3-one [R = 2,5-(H_3_CO)_2_C_6_H_3_] is the most potent analog synthesized against the breast cancer cell lines tested (MCF7, MDA-MB-231).^32^

**Scheme 7:**
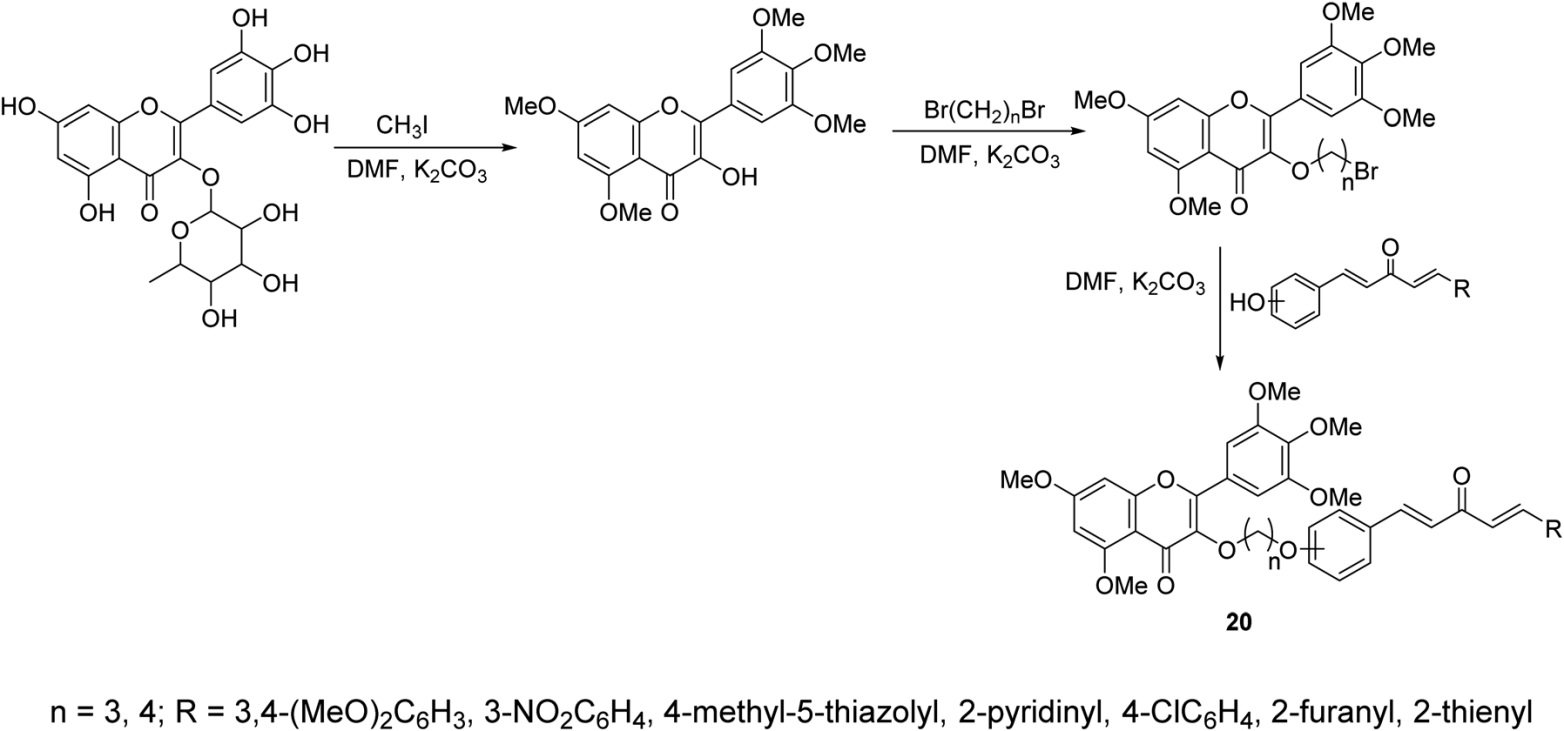
Synthetic route towards curcumin mimics conjugated chromen-4-one analogs 20.

A series of curcumin mimics conjugated with chromen-4-one analogs 20 were synthesized through reaction of (1*E*,4*E*)-1-aryl-5-(hydroxyphenyl)penta-1,4-dien-3-ones with 3-(bromoalkyloxy)-5,7-dimethoxy-2-(3,4,5-trimethoxyphenyl)-4*H*-chromen-4-one in DMF containing K_2_CO_3_ (Scheme 7). Some of the synthesized agents exhibited promising antiproliferative properties of which, 7-dimethoxy-3-(3-(2-((1*E*,4*E*)-3-oxo-5-(pyridin-2-yl)penta-1,4-dien-1-yl)phenoxy)propoxy)-2-(3,4,5-trimethoxyphenyl)-4*H*-chromen-4-one, showed the most promise against gastric cancer cell lines (SGC-7901, MGC-803) relative to doxorubicin (standard reference) with inhibitory properties against TrxR (thioredoxin reductase) observed.^33^

## 5. 3,5-Bis(ylidene)-4-piperidone, bio-active curcumin mimics

### 5.1. Antitumor active agents

**Fig. 9:**
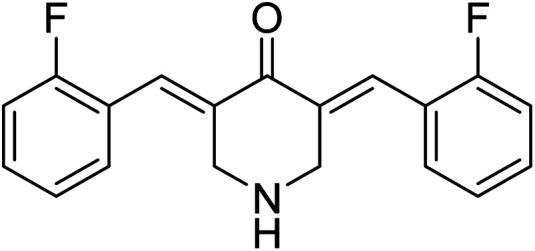
3,5-Bis((*E*)-2-fluorobenzylidene)piperidin-4-one 21.

3,5-Bis((*E*)-2-fluorobenzylidene)piperidin-4-one 21 (Fig. 9) was reported to possess antiproliferation properties against MDA-MB231 (breast) and PC3 (pancreatic) cancer cell lines with potency higher than that of curcumin. It was presumed that its mode of action due to the inhibition of intracellular pro-angiogenic transcription factor (HIF).^34^

**Fig. 10:**
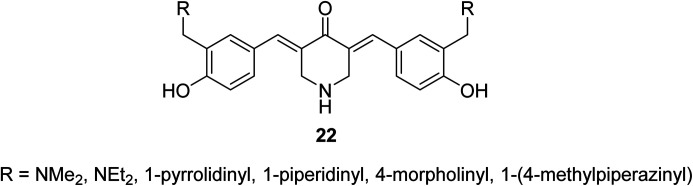
3,5-Bis(hydroxyarylidene)-4-piperidones 22 with alkylaminomethyl substituents.

3,5-Bis(4-hydroxyarylidene)-4-piperidones 22 with alkylaminomethyl substituent (Fig. 10) revealed potent antiproliferative properties against Molt 4/C8, CEM (T-lymphocyte) and L1210 (murine leukemia) cell lines (MTT assay) with higher efficacies than melphalan. Some of synthesized agents characterized as inducer of apoptosis in addition to ability for DNA fragmentation. Cleavage of poly ADP-ribose polymerase is the mode of action assumed for the bio-active agents.^35^

**Scheme 8:**
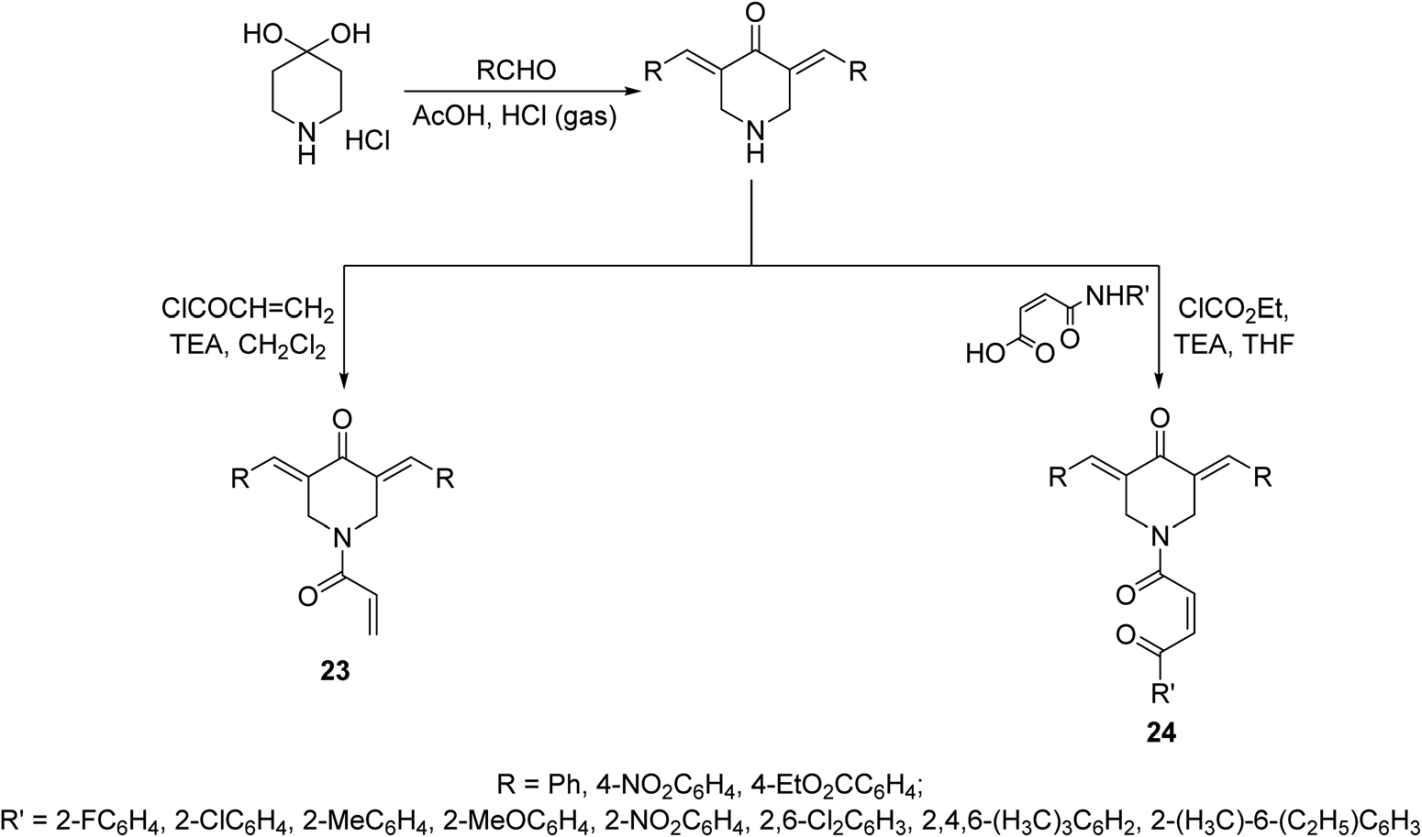
Synthetic route towards *N*-acryloyl-23 and 1-[*N*-(aryl)maleamoyl]-3,5-bis(ylidene)-4-piperidones 24.

*N*-Acryloyl-3,5-bis(ylidene)-4-piperidones 23 were synthesized through acid catalyzed condensation (AcOH/HCl gas) of 4-piperidone hydrate hydrochloride with the appropriate aldehyde followed by reaction with acryloyl chloride in CH_2_Cl_2_ containing TEA (triethylamine) at 0 °C. However, 3,5-bis(ylidene)-1-[*N*-(aryl)maleamoyl]-4-piperidones 24 were obtained through reaction of maleamic acid (generated from the reaction of maleic anhydride and aryl amine in CH_2_Cl_2_) and the corresponding 3,5-bis(ylidene)-4-piperidones in THF (tetrahydrofuran) containing ethyl chloroformate and TEA (Scheme 8). Most of the synthesized agents exhibited cytostatic properties against human Molt4/C8, CEM (T-lymphocytes) and L1210 (leukemic) cell lines with higher potency than curcumin and melphalan (standard reference, used in chemotherapeutical combination for chronic leukemia and wide range of malignancies). Safety profile was achieved *in vitro* (WI-38, human fibroblasts cells) and *in vivo* (mice) testing. Some of the synthesized agents showed potent inhibitory properties of topoisomerase IIα. This enzyme facilitates DNA replication by preventing the buildup of supercoils during replication fork progression. Inhibitors of this enzyme result in the generation of multiple DNA strand breaks, arrest cell division and eventually lead to apoptosis. As such numerous topoisomerase inhibitors are currently used in clinics as chemotherapy for numerous malignancies. The synthesized agents also revealed antioxidant properties.^36^

**Scheme 9:**
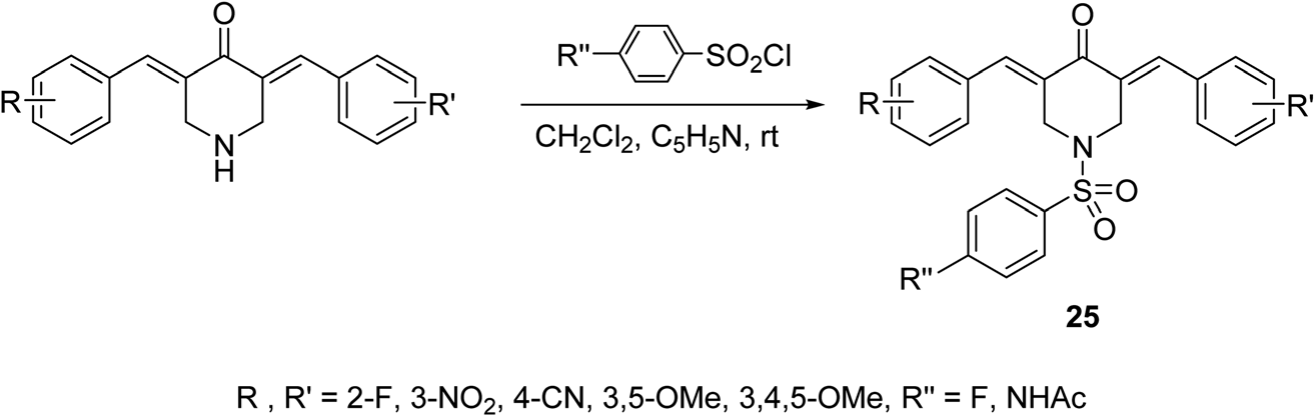
Synthetic route towards *N*-arylsufonyl-3,5-bis(arylidene)-4-piperidones 25.

A series of *N*-arylsulfonyl-3,5-bis(arylidene)-4-piperidones 25 were synthesized through base-catalyzed arylsulfonation (catalytic amount of pyridine in CH_2_Cl_2_ at room temperature) of the corresponding *N*-unsubstituted piperidones (Scheme 9). The synthesized agents showed anti-inflammatory properties supported by the inhibition of IL-6 and TNF-α in RAW264.7 cells induced by lipopolysaccharide (LPS of Gram-negative bacteria). Promising antiproliferation properties were also mentioned by some of the synthesized agents against liver (HepG2, SMMC-7721, QGY-7703) cancer cell lines (MTT assay) with induction of apoptosis. Association of chronic inflammation with cancer progression especially, hepatic cancer is the rational for investigation of anti-inflammatory and antiproliferation properties of the synthesized agents.^37^

**Fig. 11:**
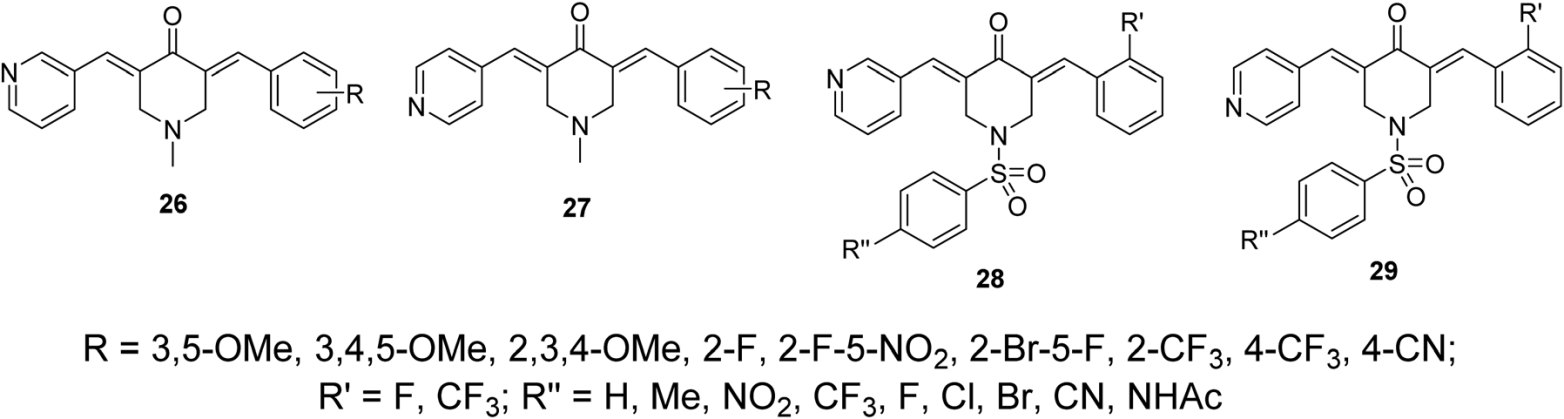
Dissymmetric pyridine-containing 3,5-(arylidene)-4-piperidones 26–29.

Other sets of dissymmetric pyridine-containing 3,5-bis(arylidene)-4-piperidones 26–29 were also reported (Fig. 11) with anti-inflammatory and anti-hepatoma properties similar to compounds 25.^38^

**Scheme 10:**
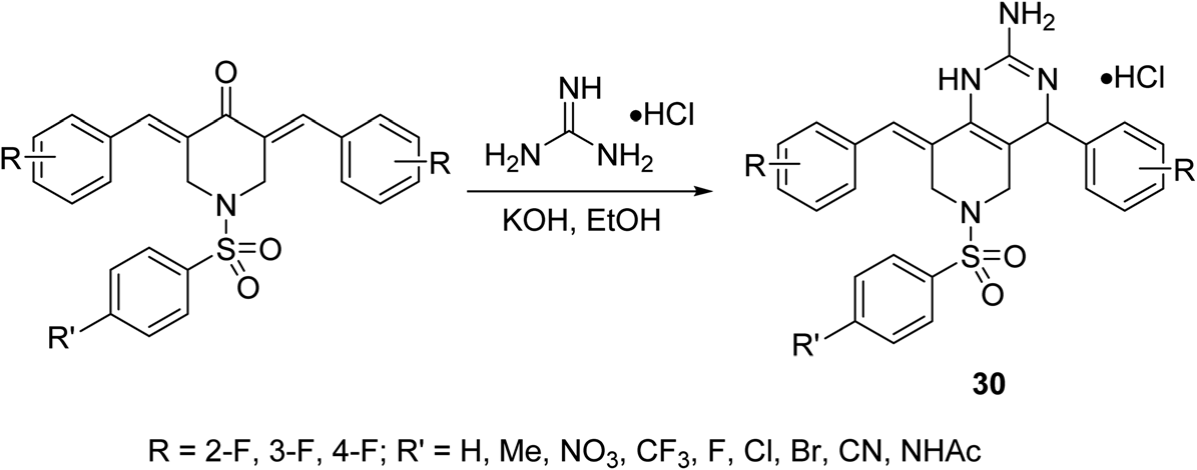
Synthetic route towards pyrido[4,3-*d*]pyrimidines 30.

Pyrido[4,3-*d*]pyrimidines 30 were synthesized through reaction of the corresponding fluoro-containing *N*-arylsulfonyl-3,5-bis(arylidene)-4-piperidones with guanidine hydrochloride in ethanolic KOH (Scheme 10). Antiproliferative properties were observed by the targeted agents against a variety of hepatocellular carcinoma cells (HepG2, SMMC-7721) using MTT assay. Inhibition of the nuclear translocation of NF-κB induced by TNF-α or LPS supports the anti-inflammatory properties of these compounds, considering that NF-κB is the signal pathway connecting the chronic inflammation and hepatocellular carcinoma.^39^

**Scheme 11:**
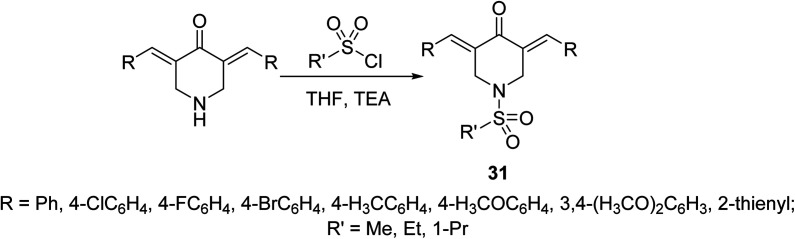
Synthetic route towards 1-(alkylsulfonyl)-3,5-bis(ylidene)-4-piperidinones 31.

Potent antiproliferative agents against HCT116 (colon) and A431 (skin/squamous) cancer cell lines were exhibited by 1-(alkylsulfonyl)-3,5-bis(ylidene)-4-piperidinones 31 (Scheme 11) relative to 5-fluorouracil (approved drug for colon, breast and skin cancers). Some of the synthesized agents also showed high potency against MCF7 (breast) and A549 (lung) cancer cell lines (relative to 5-fluorouracil and doxorubicin) with minimal cytotoxicity against RPE1 (non-cancer, retinal pigment epithelial) cell line. The synthesized agents exerted their mode of action *via* inhibitory properties of topoisomerase IIα which is the enzyme responsible for breaking double strand DNA helix during DNA replication, transcription and repairing.^40^

**Scheme 12:**
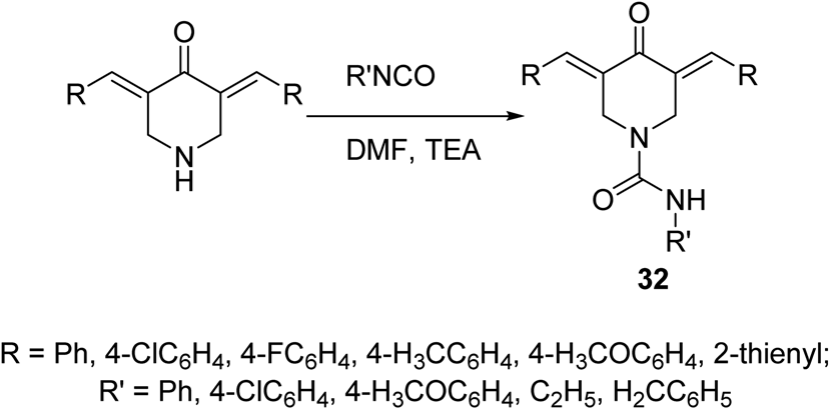
Synthetic route towards 3,5-bis(ylidene)-*N*-substituted-4-piperidinone-1-carboxamides 32.

A set of 4-piperidone-1-carboxamides 32 were synthesized *via* reaction of isocyanate with the appropriate *N*-unsubstituted 3,5-diylidene-4-piperidine in DMF in the presence of TEA (Scheme 12). Most of the synthesized agents revealed high potency against HCT116 (colon), MCF7 (breast) and A431 (skin/squamous) cancer cell lines with higher efficacy than that of 5-fluorouracil and safe behavior against non-cancer (RPE1) cell line. The synthesized agents revealed topoisomerase II-α inhibitory properties supporting their mode of action.^41^

**Scheme 13:**
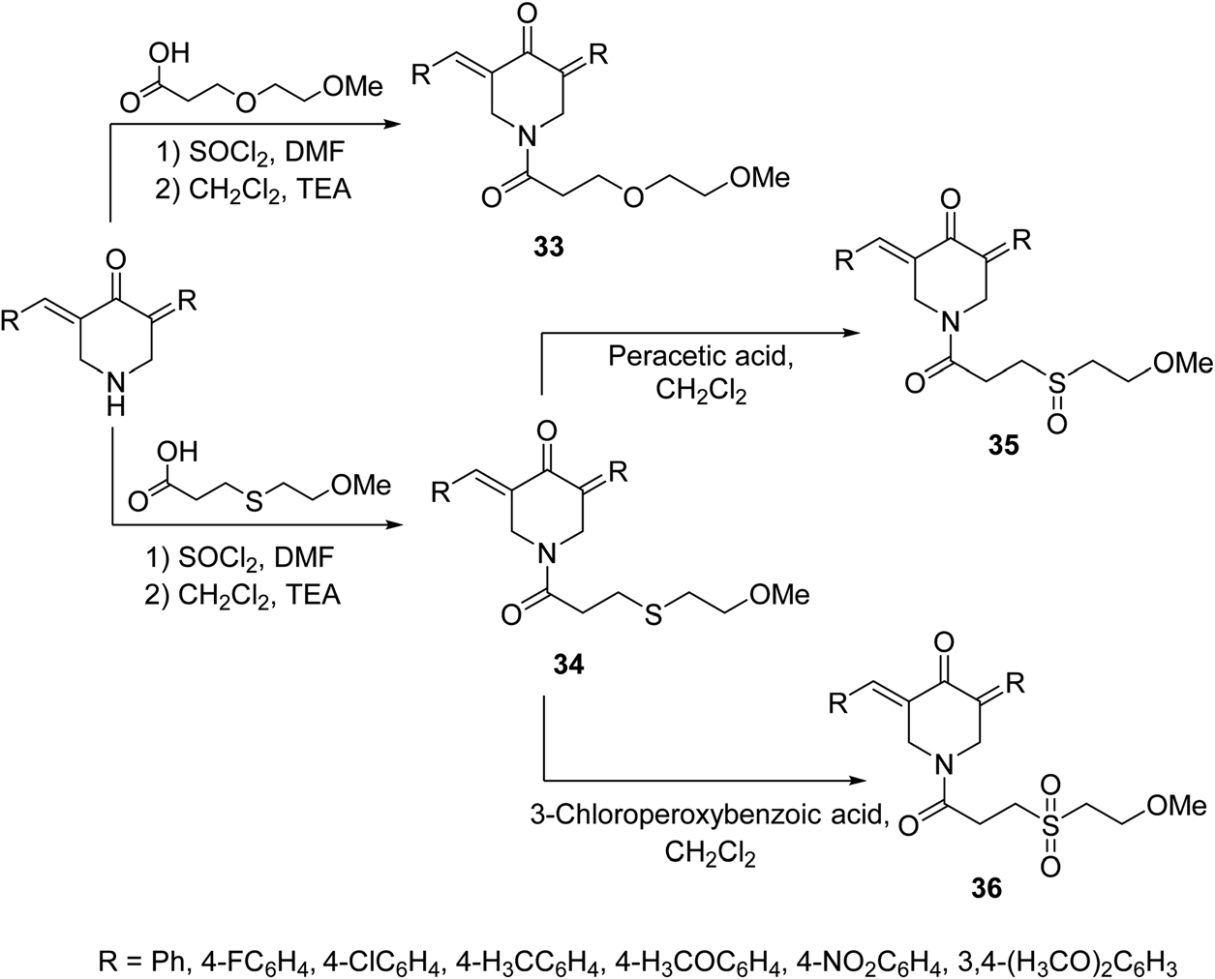
Synthetic route towards 1-[3-(2-methoxyethyloxy)propionyl]-4-piperidones 33 and their thio analogs 34–36.

A set of 1-[3-(2-methoxyethyloxy)propionyl]-4-piperidones 33 and their thio-analogs 34 were synthesized through reaction of the acyl chloride with the appropriate 3,5-bis(ylidene)-4-piperidone in CH_2_Cl_2_ containing triethylamine. The sulfinyl-35 and sulfonyl-36 derivatives were obtained through oxidation of compounds 34 with peracetic acid and 3-chloroperoxybenzoic acid in CH_2_Cl_2_, respectively (Scheme 13). Antiproliferative properties were observed by the synthesized agents against human Molt 4/C8, CEM (T-lymphocyte) and L1210 (murine leukemia) cell lines with safer behavior towards non-malignant cells. Some of the synthesized agents demonstrated PARP1 [poly(ADP-ribose)polymerase 1] cleavage, a characteristic hallmark of apoptosis. PARP1 is capable of repairing DNA single-stranded breaks, thus compounds that induce PARP1 cleavage can prevent DNA replication and are useful in cancer chemotherapy.^42^

**Scheme 14:**
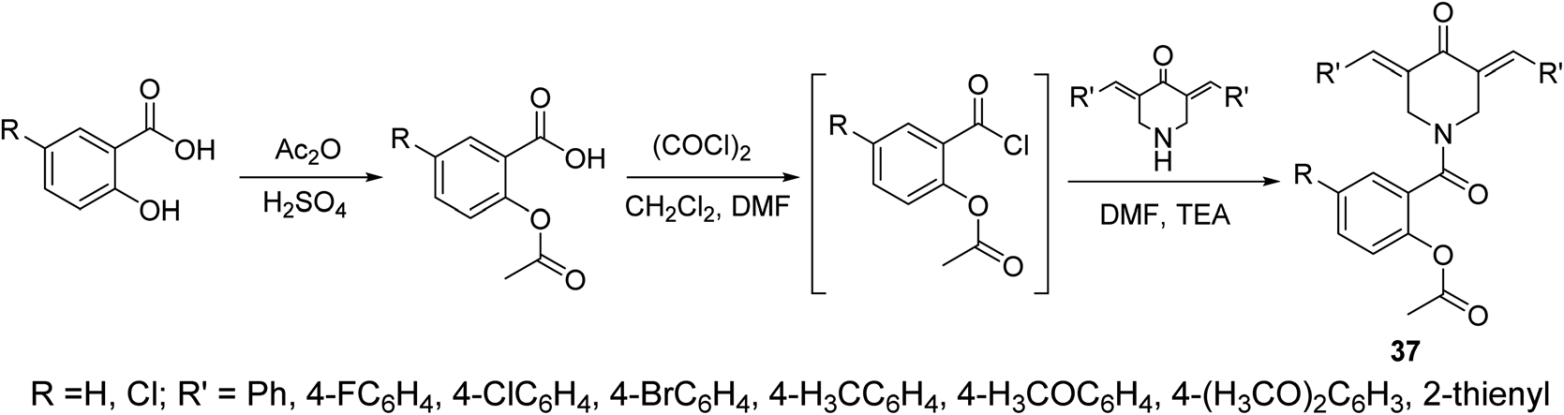
Synthetic route towards piperidone-salicylate conjugates 37.

The piperidone-salicylate conjugates 37 were synthesized through dehydrohalogentation of acetylsalicylic acid chloride with the appropriate piperidone in DMF containing TEA as basic catalyst (Scheme 14). Potent antiproliferation properties were noticed by the synthesized conjugates against A431 (squamous skin), HCT116 (colon) and MCF7 (breast) cancer cell lines (MTT assay) with comparable efficacies to that of 5-fluorouracil and sunitinib (standard references). Multi-targeted inhibitory properties were observed against VEGFR-2 (vascular endothelial growth factor receptor-2) and EGFR (epidermal growth factor receptor) in both MCF7 (breast) and HCT116 (colon) cancer cells. Enhanced COX-1 and COX-2 (cyclooxygenase-1 and -2) inhibitory properties were also revealed by the synthesized agents than that of aspirin supporting their anti-inflammatory properties. Selective inhibition was noticed towards COX-2 compared to COX-1. Additionally, some of the synthesized agents revealed antiviral properties against SARS-CoV-2 (respiratory syndrome coronavirus 2) which is the responsible infectious microorganism of COVID-19 (corona virus disease 2019) pandemic.^43^

**Scheme 15:**
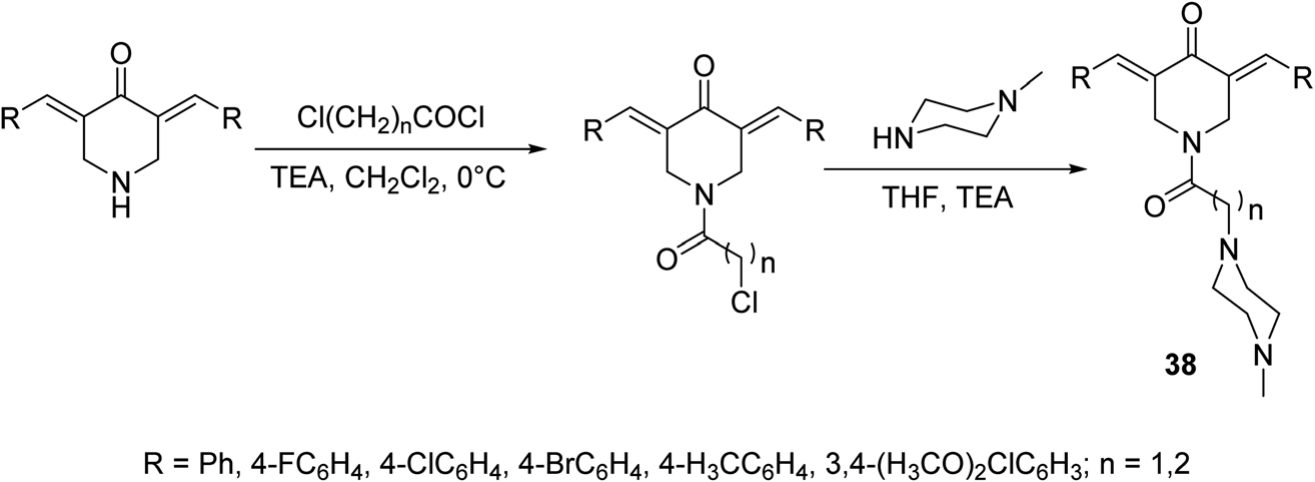
Synthetic route towards 3,5-di[(*E*)-arylidene]-1-[3-(4-methylpiperazin-1-yl)alkyl]piperidin-4-ones 38.

A set of antiproliferative active agents 3,5-di[(*E*)-arylidene]-1-[3-(4-methylpiperazin-1-yl)alkyl]piperidin-4-ones 38 were prepared through acylation of the appropriate 3,5-bis(ylidene)-4-piperidones followed by dehydrohalogenation *via* reaction with *N*-methylpiperazine (Scheme 15). Potent antiproliferative properties were exhibited against HCT116 (colon) and MCF7 (breast) cancer cell lines relative to sunitinib and 5-fluorouracil (standard references). Dual inhibitory properties were observed against human topoisomerase I and IIα. However, with higher efficacy against topoisomerase IIα than I. Promising anti-SARS-CoV-2 properties were also revealed relative to favipiravir (standard reference) during the VERO-E6 standard technique. Lack of cytotoxicity against normal RPE1 and VERO-E6 cells supports the possibility that these compounds may be potential drug candidates.^44^

**Scheme 16:**
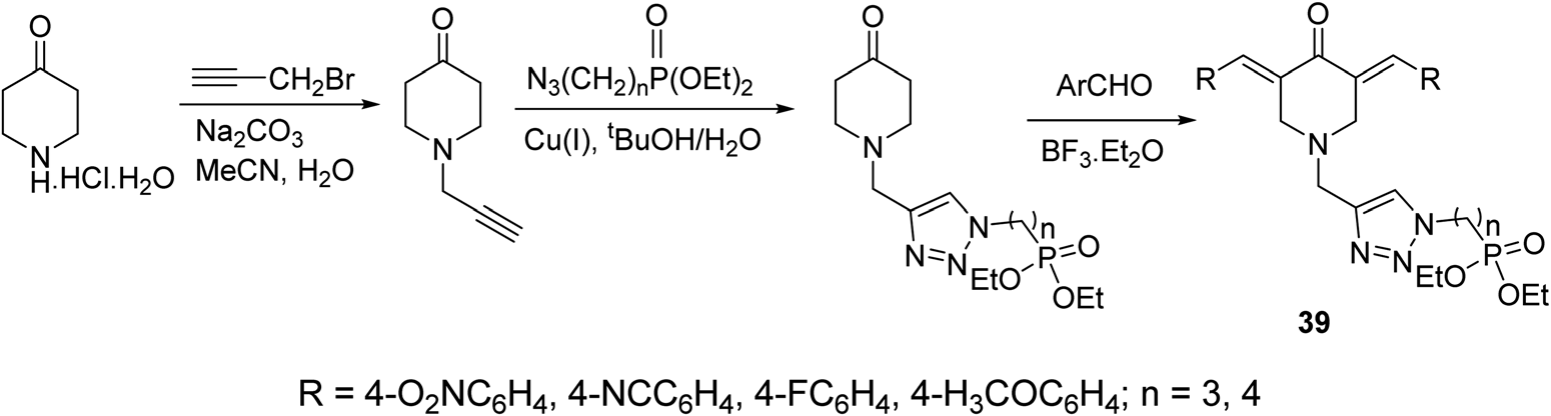
Synthetic route towards 3,5-bisarylidene-4-piperidones connected with 1,2,3-triazolyl heterocycle bearing phosphonate group 39.

A series of 3,5-bis(arylidene)-4-piperidones connected with 1,2,3-triazolyl heterocycle bearing phosphonate group 39 were obtained *via* Aldol condensation (Et_2_O·BF_3_) (Scheme 16). Promising antitumor properties were observed against HCT116 (colon) and MCF7 (breast) cancer cell lines (relative to doxorubicin, reference standard) with limited toxicity against normal (HEF) cells.^45^

**Scheme 17:**
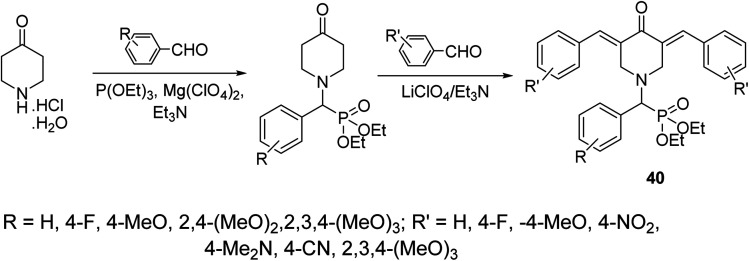
Synthetic route towards 3,5-bis(arylidene)-4-piperidones attached to diethyl[(aryl)methyl]phosphonate moiety 40.

A series of 3,5-bis(arylidene)-4-piperidones attached to diethyl[(aryl)methyl]phosphonate moiety 40 were synthesized *via* reaction of α-amino(aryl)methyl phosphonates (obtained through Kabachnik–Fields reaction of triethyl phosphite, appropriate aldehyde and 4-piperidone hydrochloride monohydrate) with aromatic aldehyde in the presence of LiClO_4_/Et_3_N (Lewis acid) (Scheme 17). Noticeable antiproliferative properties were observed against a variety of human tumor cell lines (RD “rhabdomyosarcoma”, PC3 “pancreatic”, HCT116 “colon”, and MCF7 “breast”) cell lines relative to doxorubicin and daunorubicin (reference standards).^46^

**Scheme 18:**
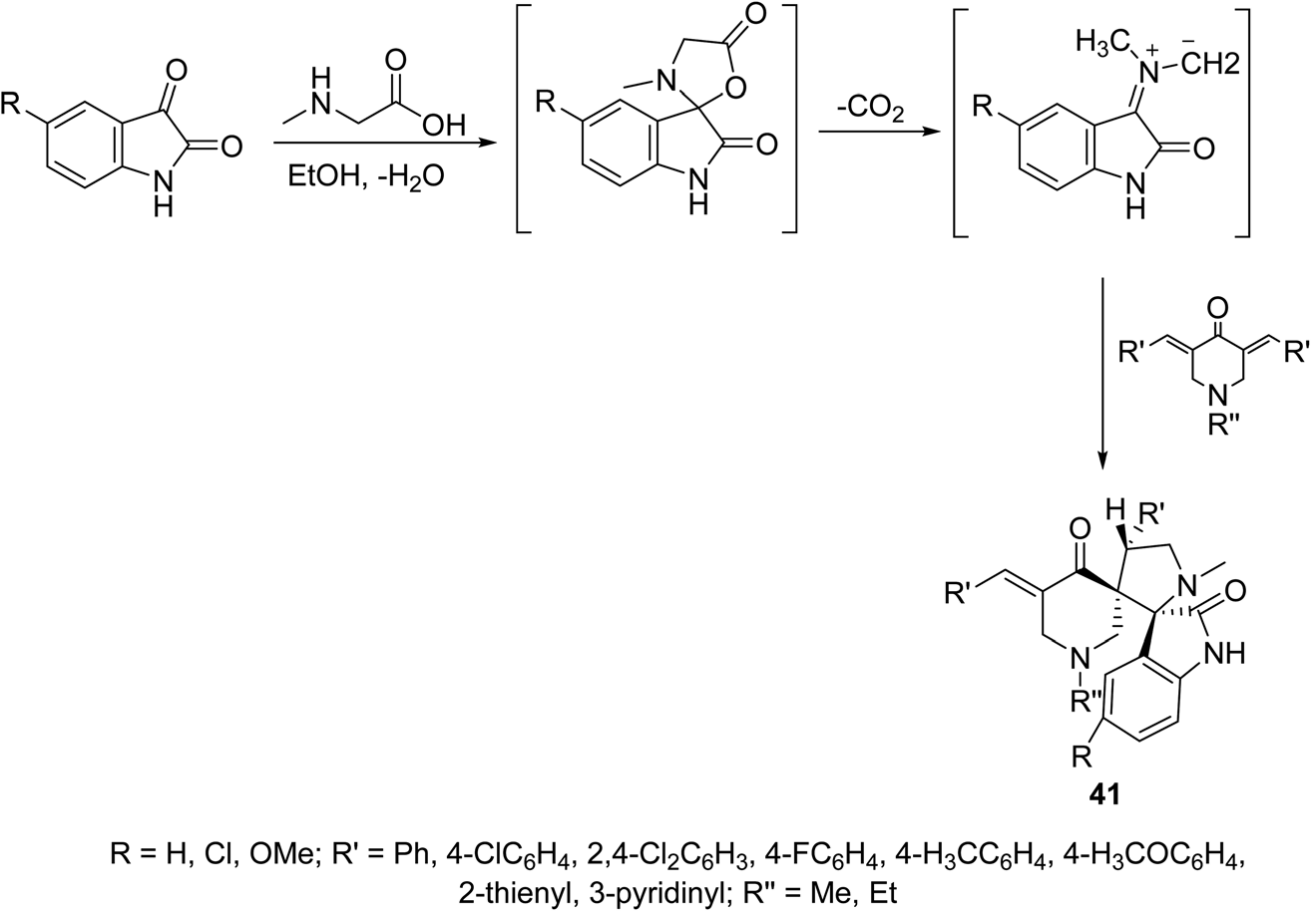
Synthetic route towards dispiro[3*H*-indole-3,2′-pyrrolidine-3′,3′′-piperidines] 41.

A series of dispiro[3*H*-indole-3,2′-pyrrolidine-3′,3′′-piperidines] 41 were synthesized through dipolar cycloaddition of the appropriate 3,5-bis(ylidene)-4-piperidones with azomethine ylide (which obtained *in situ* through reaction of sarcosine with isatins) (Scheme 18). The stereochemical structure of 41 was established through single crystal X-ray studies. Many of the synthesized agents showed promising antiproliferation properties against HeLa (cervical), MCF7, T-47D (breast), HepG2 (liver) and HCT116 (colon) carcinoma cell lines (SRB technique) relative to that of doxorubicin and cisplatin (standard references). QSAR (CODESSA-Pro, CODESSA III software) and 3D-pharmacophoric (Discovery Studio 2.5) studies discussed the biological properties observed, optimized molecular models and exhibited the descriptors/chemical features necessary for bio-observations.^47–49^

**Fig. 12:**
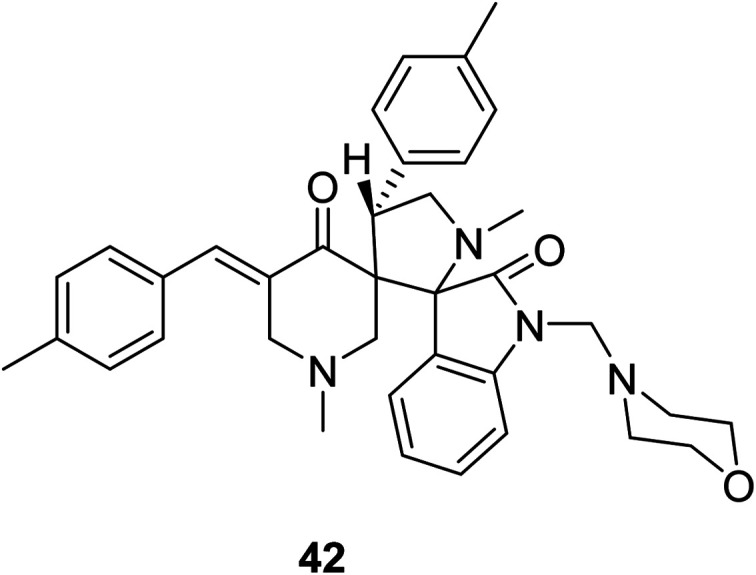
Dispiro-analog bearing 1-[(4-morpholinyl)methylene] function 42.

Dispiro-analog bearing a 1-[(4-morpholinyl)methylene] group 42 was also synthesized through azomethine dipolar cycloaddition reaction with the appropriate piperidone (Fig. 12). Quantum chemical calculations [DFT/B3LYP, 6-31G(d,p)] determined the stereochemical structure. Promising antitumor properties were observed against diverse human cancer cell lines (National Cancer Institute screening program).^50^

### 5.2. Cholinesterase inhibitors

**Fig. 13:**
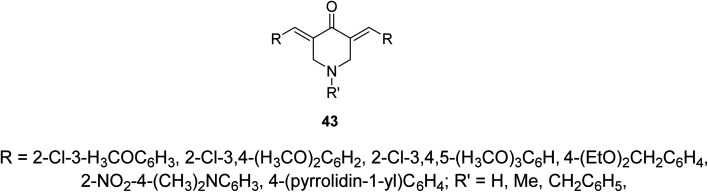
3,5-Bis(ylidene)-4-piperidones of cholinesterase inhibitory properties 43.

Alzheimer's disease is a neurodegenerative disease comprising the main cause of dementia particular in elder people. One of the most common methods for treatment is the elevation of acetylcholine levels in the brain. Acetylcholinesterase (AChE) and butyrylcholinesterase (BChE) are two enzymes that exist in the central nervous system capable of hydrolyzing acetylcholine (neurotransmitter at cholinergic synapses). For this reason compounds with inhibitory properties against these enzymes are valuable for controlling progress of this disease. A series of 3,5-bis(ylidene)-4-piperidones 43 (Fig. 13) showed promising inhibitory properties against AChE and BChE (Ellman's method) compared to Tacrine and Donepezil (standard references).^51,52^

**Scheme 19:**
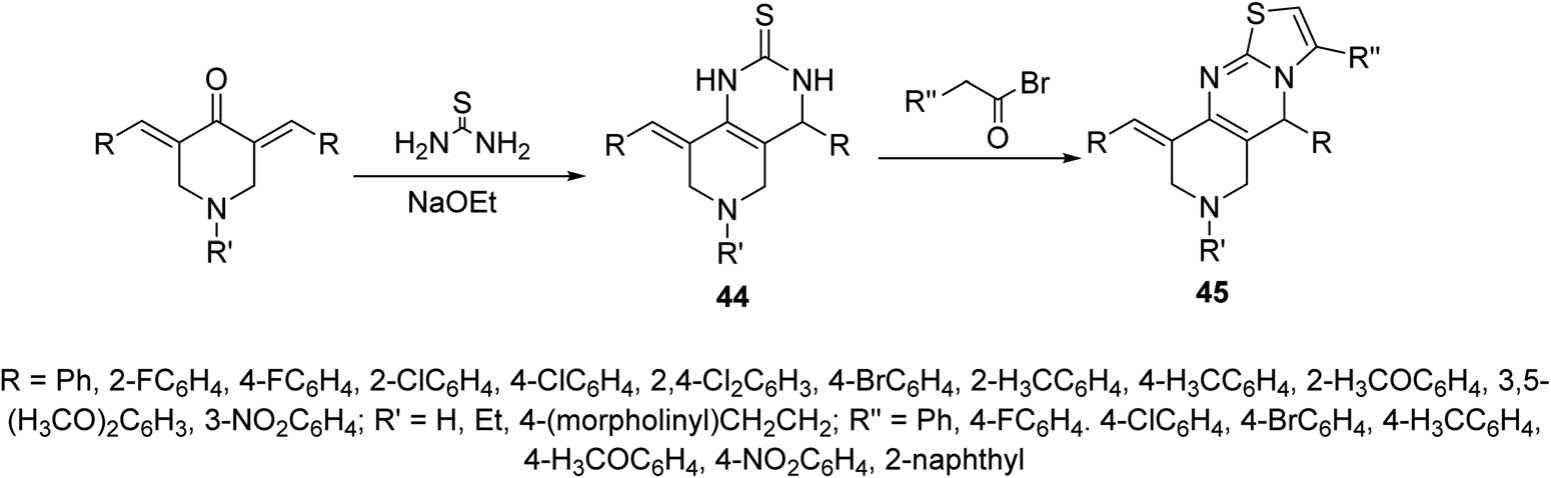
Synthetic route towards pyrido[4,3-*d*]pyrimidine 44 and pyrido[4,3-*d*]thiazolo[3,2-*a*]pyrimidines 45.

Pyrido[4,3-*d*]pyrimidines 44 obtained through reaction of 3,5-bis(ylidene)-4-piperidones with thiourea in presence of NaOEt. Reaction of 44 with phenacyl bromides afforded the formation of pyrido[4,3-*d*]thiazolo[3,2-*a*]pyrimidines 45. Alternatively, 45 could be obtained directly through domino reaction of the appropriate piperidone, thiourea and phenacyl bromide in ionic liquid (1-butyl-3-methylimidazolium bromide “[bmim]Br”) under microwave irradiation (Scheme 19). Promising AChE and BChE inhibitory properties were observed by some of the synthesized agents 44 and 45 (Ellman's method) relative to Galantamine.^53–55^

**Scheme 20:**
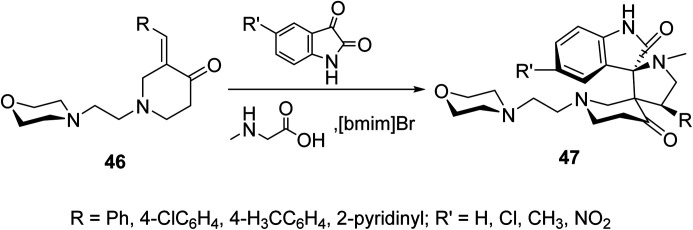
Synthetic route towards spiropyrrolidines 47.

Spiropyrrolidines 47 were synthesized *via* [3 + 2]-dipolar cycloaddition reaction of 3-(ylidene)-*N*-substituted-4-piperidones 46 with azomethine ylides formed from condensation of the appropriate isatin and sarcosine in presence of ionic liquid “[bmim]Br” (Scheme 20). Promising AChE and BChE inhibitory properties were observed by some of the synthesized agents (Ellman's method) compared with Galantamine (reference standard).^56^

**Scheme 21:**
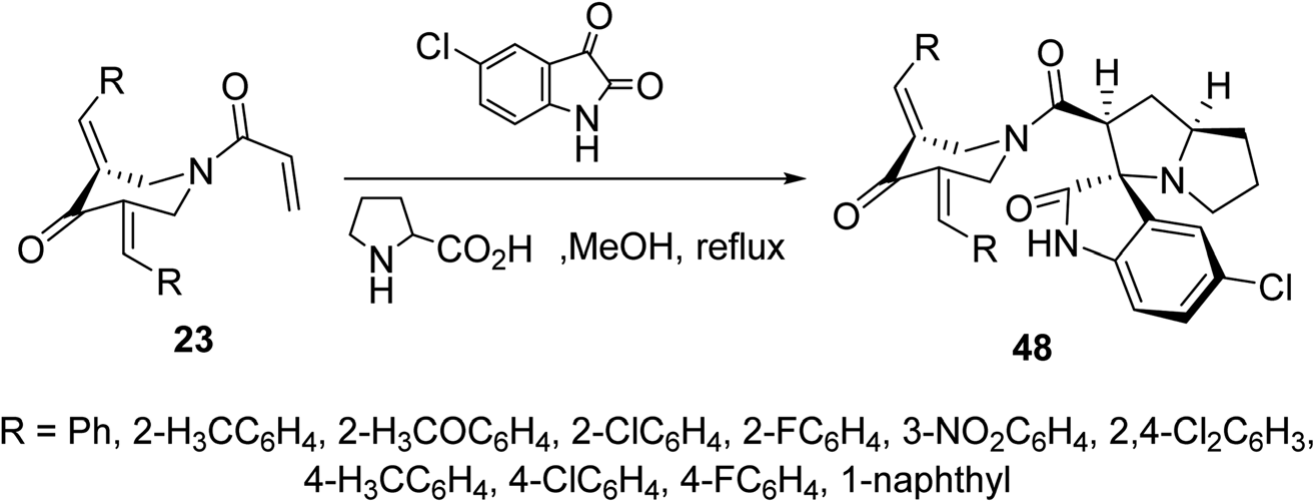
Synthetic route towards spiro-heterocycles 48.

Cycloaddition reaction of azomethine ylide (formed from condensation of 5-chloroisatin and l-proline) with *N*-acryloyl-3,5-bis(ylidene)-4-piperidones 23 afforded the corresponding spiro-heterocycles 48*via* reaction with the acryloyl linkage rather than the exocyclic ylidene olefinic linkage (Scheme 21). Stereochemical structure of 48 was supported by single crystal X-ray studies. AChE and BChE properties were exhibited by the synthesized agents in comparison with Galantamine (standard reference).^57^

**Scheme 22:**
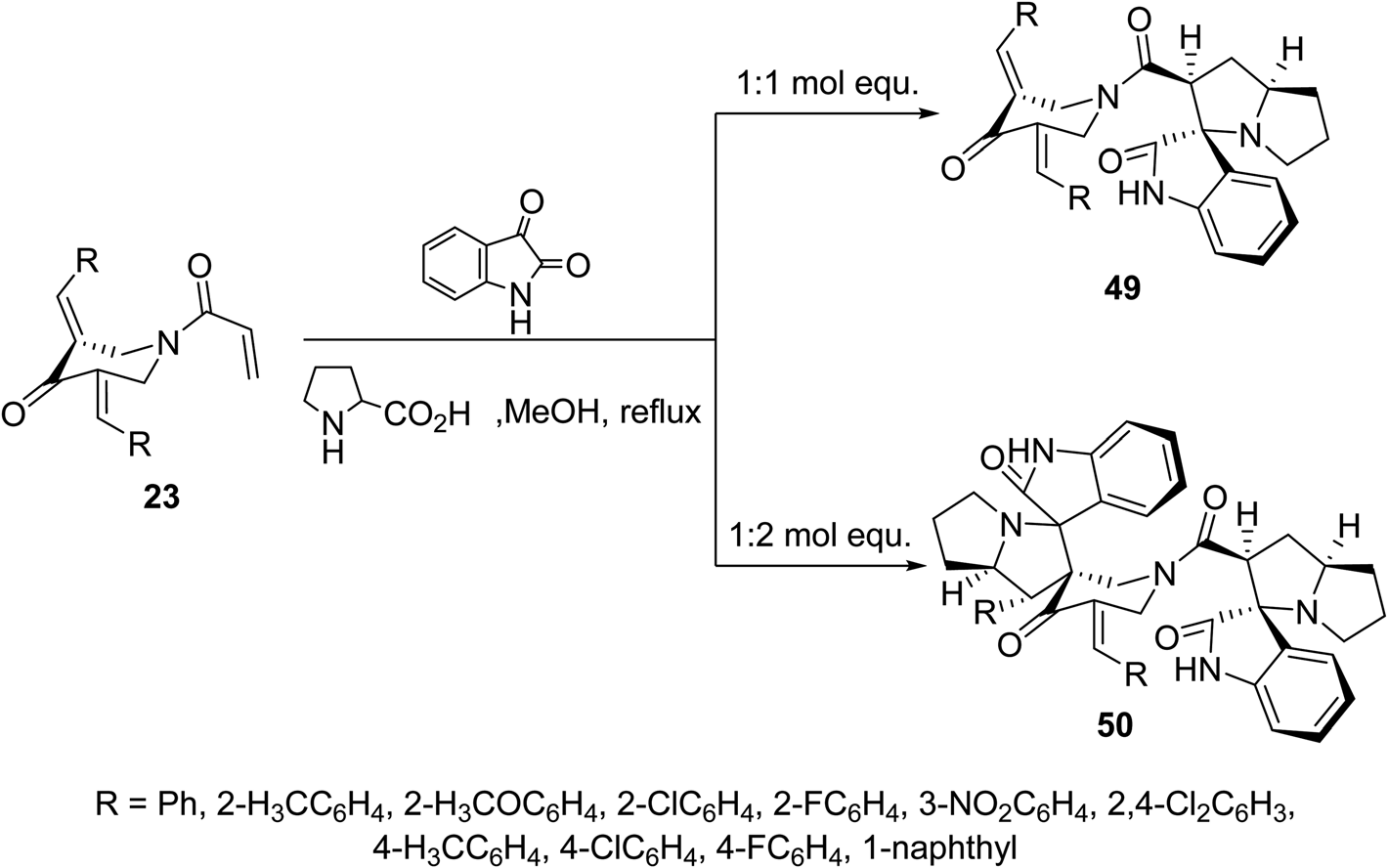
Synthetic route towards mono-spiro-49 and bis-spiro-heterocycles 50.

Additionally, azomethine cycloaddition reaction (formed from isatin and l-proline) with *N*-acryloyl-3,5-bis(ylidene)-4-piperidones 23 in refluxing MeOH in equimolar values afforded the mono-spiro-heterocycles 49 in a similar manner to that of the aforementioned formation of 48. However, reaction of azomethine ylide in double folds amount (two molar equivalents) to that of piperidones 23, the bis-spiro-heterocycles 50 were obtained due to double cycloaddition reactions with both acryloyl and ylidene linkages (Scheme 22). Both compounds 49 and 50 revealed AChE and BChE inhibitory properties (Ellman's method) and some of them showed potency comparable to that of Galantamine.^58^

**Scheme 23:**
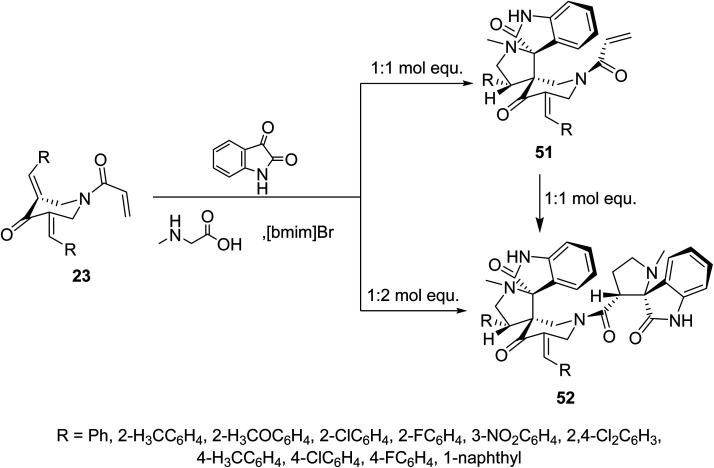
Synthetic route towards mono-spiro-51 and bis-spiro-heterocycles 52.

Meanwhile, ionic liquid “[bmim]Br” mediated cycloaddition reaction of azomethine ylide derived from isatin and sarcosine with *N*-acryloyl-3,5-bis(ylidene)-4-piperidones 23 afforded the mono-spiro-pyrrolidines 51 due to cycloaddition reaction with the exocyclic ylidene linkage. However, reaction of the azomethine ylide with 23 in 2 : 1 molar value equivalent afforded the bis-spiro-pyrrolidines 52. The difference in observations of this reaction to that mentioned in Scheme 22 is attributed to the different reactant azomethine ylide derived from diverse amino acid (sarcosine and l-proline). Additionally, bis-spiro-pyrrolidines 52 were obtained from mono-spiro-pyrrolidines 51 by reaction with another mol equivalent of the azomethine ylide (Scheme 23). Both mono-spiro-51 and bis-spiro-pyrrolidines 52 revealed noticeable AChE and BChE inhibitory properties and some of them exhibited potency comparable to that of Galantamine.^59^

**Scheme 24:**
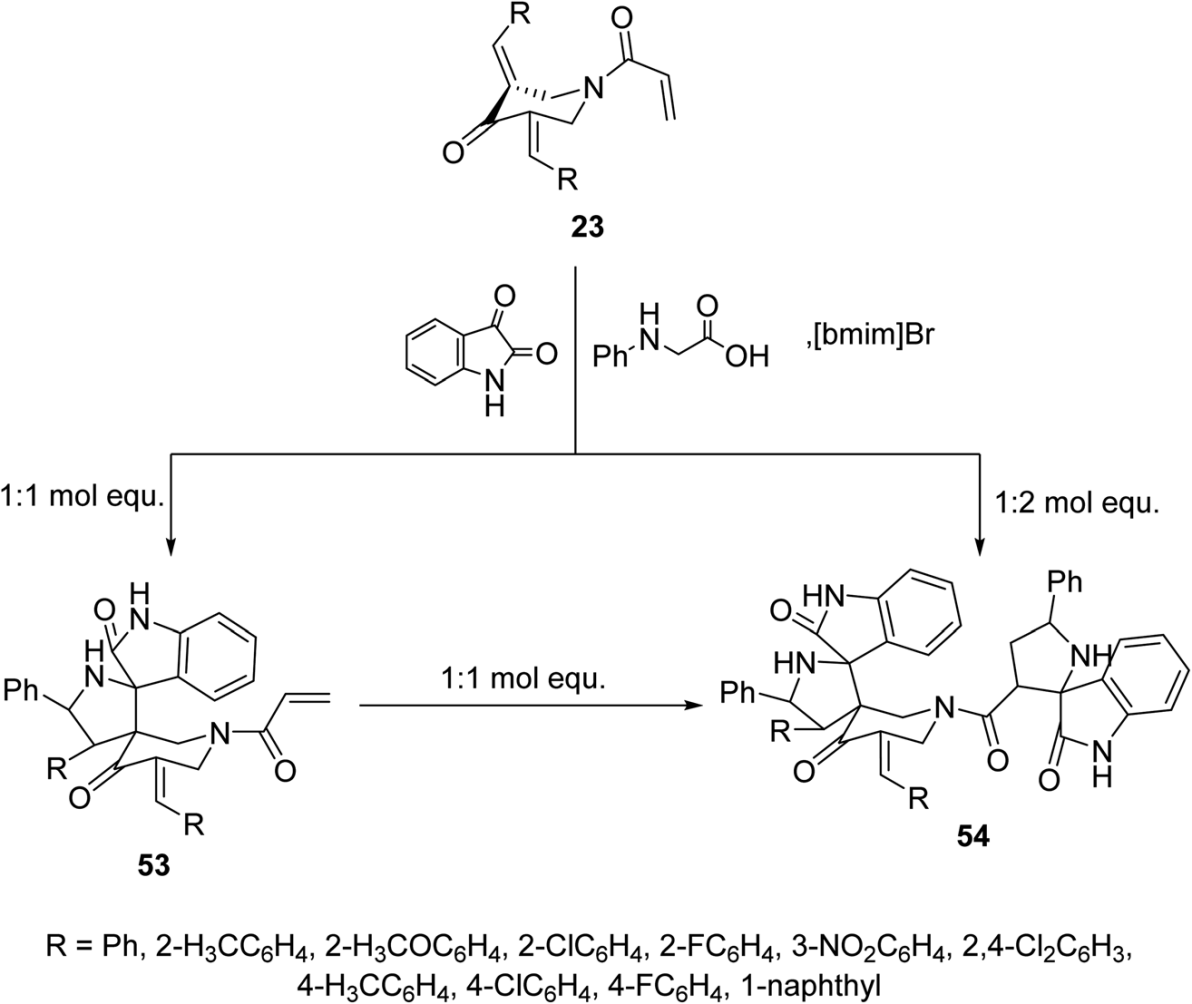
Synthetic route towards mono-spiro-53 and bis-spiro-heterocycles 54.

Similarly, reaction of *N*-acryloyl-3,5-bis(ylidene)-4-piperidones 23 with azomethine ylide generated from phenylglycine and isatin (in 1 : 1 molar equivalent) in ionic liquid “[bmim]Br” medium afforded the mono-spiro-pyrrolidines 53 due to cycloaddition reaction with the exocyclic ylidene linkage. However, reaction of the azomethine ylide with 23 in 2 : 1 molar value equivalent afforded the bis-spiro-pyrrolidines 54. Reaction of 53 with another mol equivalent of azomethine ylide also afforded the bis-spiro-pyrrolidines 54 (Scheme 24). AChE and BChE inhibitory properties were shown by the mono-spiro-53 and bis-spiro-pyrrolidines 54 and some of them exhibited potency comparable to that of Galantamine.^60^

**Scheme 25:**
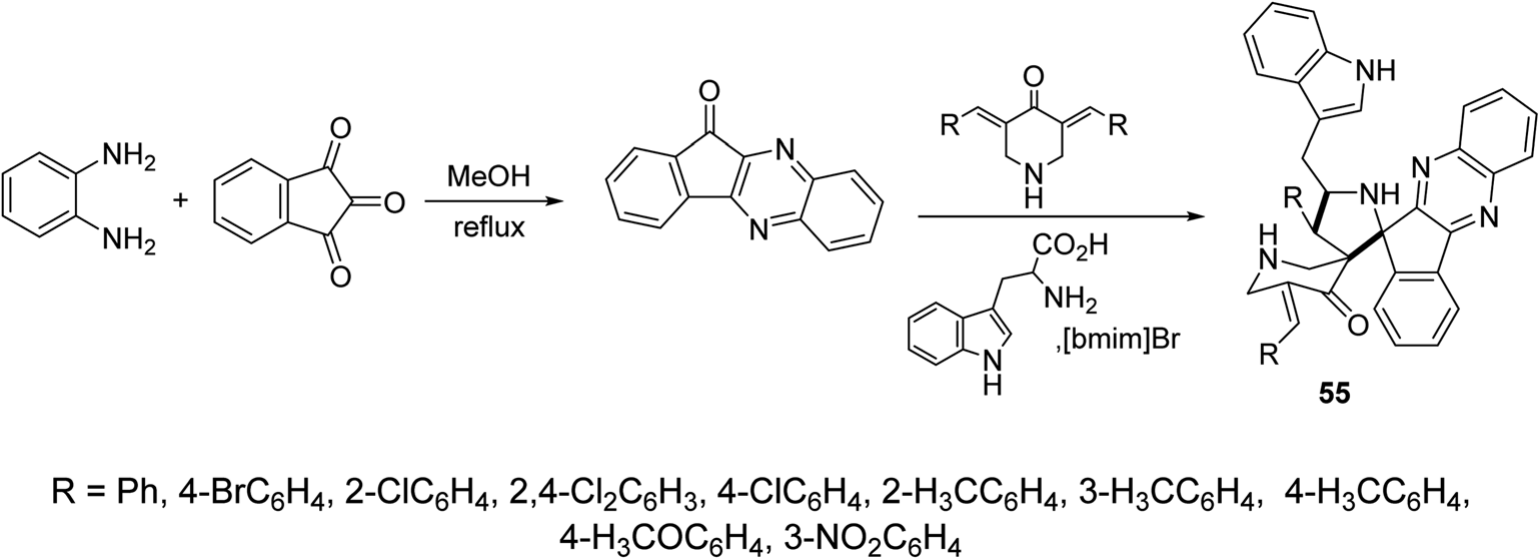
Synthetic route towards dispiropyrrolidines 55.

Indeno[3,2-*b*]quinoxalin-11-one were synthesized through condensation of ninhydrin and *o*-phenylenediamine in refluxing methanol. Ionic liquid mediated “[bmim]Br” multicomponent [3 + 2]-dipolar cycloaddition reaction of azomethine ylide (obtained from the condensation of l-tryptophan and indeno[3,2-*b*]quinoxalin-11-one) with 3,5-bis(ylidene)-4-piperidine afforded the dispiropyrrolidines 55 (Scheme 25). AChE and BChE inhibitory properties were shown by the synthesized dispiropyrrolidines 55 relative to Galantamine.^61^

### 5.3. Anti-inflammatory active agents

**Scheme 26:**
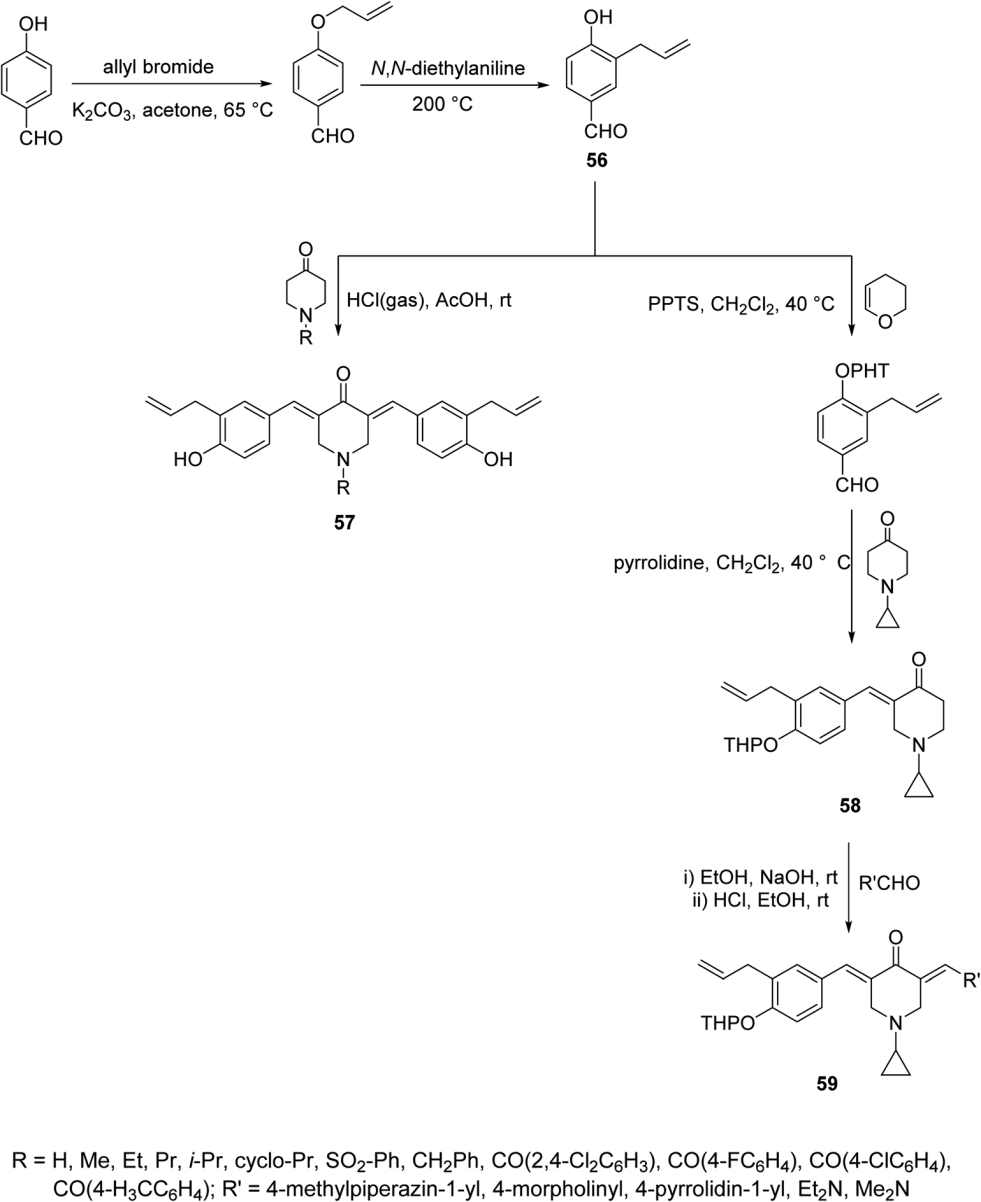
Synthetic route towards (allylated-benzylidene)-4-piperidones 57, 59.

Acute lung injury is life threatening and usually associated with acute inflammatory factors IL-6, IL-1β (interleukin) and TNF-α (tumor necrosis factor). The 3,5-bis(3-allyl-4-hydroxybenzylidene)-4-piperidones 57 were synthesized through acid-catalyzed condensation [HCl(gas)/AcOH] of 4-piperidones with 3-allyl-4-hydroxybenzaldehyde (56). Meanwhile, 3-aryliden-4-piperidone 59 was obtained *via* condensation of the 3-(ylidene)-1-(cyclopropyl)-4-piperidone 58 (O-THP protected benzaldehyde, obtained from reaction of 56 with 3,4-dihydro-2*H*-pyran) with various aldehydes (base-catalyzed reaction, NaOH/EtOH) (Scheme 26). Piperidones 57 and 59 revealed inhibitory properties against IL-6 and TNF-α supporting the possibility as a potential treatment to prevent inflammation associated with lung injury.^62^

**Fig. 14:**
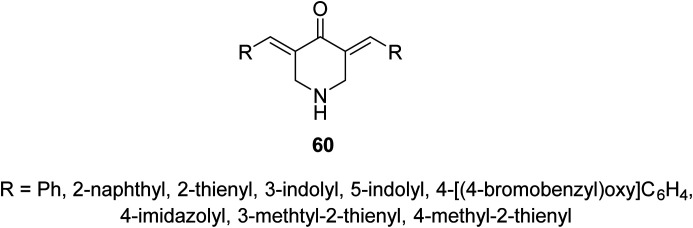
3,5-Bis(ylidene)-4-piperidones 60 of anti-inflammatory properties.

Anti-inflammatory properties of 3,5-bis(ylidene)-4-piperidones 60 (Fig. 14) were supported by the *in vivo* carrageenin-induced paw oedema of rats (i.p., 0.01 mmol per kg body weight). Some of the tested agents revealed higher efficacy than that of indomethacin (non-steroidal anti-inflammatory drug) after 3.5 h of administration.^63^

**Scheme 27:**
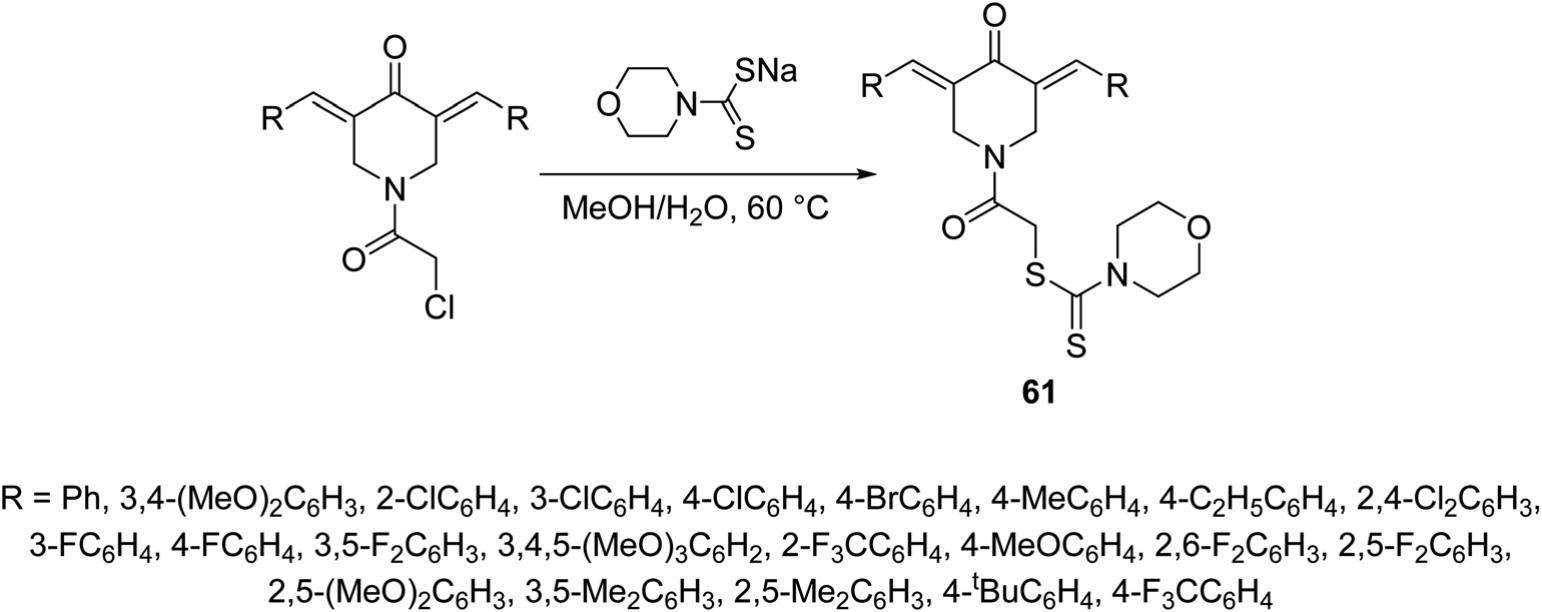
Synthetic route towards 3,5-[bis(ylidene)-4-piperidone-1-yl]-2-oxoethylmortholine-4-carbodithioates 61.

2-{3,5-[Bis(ylidene)-4-oxopiperidin-1-yl]}-2-oxoethylmorpholine-4-carbodithioates 61 where synthesized through reaction of sodium morpholine-4-carbodithioate with the appropriate 1-chloroacetyl-3,5-bis(ylidene)-4-piperidones in methanol–water (1 : 1) at 60 °C (Scheme 27). Some of the synthesized 61 revealed promising down-regulation properties of TNF-α-induced NF-κB activation in KBM5 cell. NF-κB possesses a dual role due to its capability to regulate many growth factors and cytokines responsible for anti-apoptosis, angiogenesis and metastasis. Suppression of NF-κB is an important therapeutical pathway against cancer and inflammation of cells.^64^

**Scheme 28:**
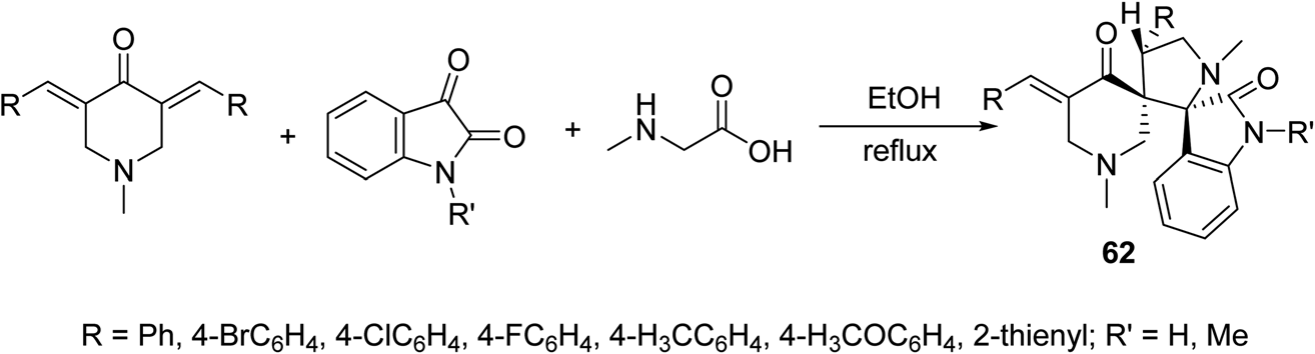
Synthetic route towards dispiro-heterocycles 62 of anti-inflammatory properties.

Dispiro-heterocyles 62 were synthesized by the azomethine ylide [3 + 2]-cycloaddition reaction with 3,5-bis(ylidene)-4-piperidones in refluxing EtOH (Scheme 28). Single crystal X-ray studies supported the structure. Anti-inflammatory properties of the synthesized agents were established by the carrageenin-induced paw edema method of rats (i.p., 50 mg per kg body weight). Some of the synthesized agents revealed higher anti-inflammatory potency than that of indomethacin after 4 h of administration.^65^

### 5.4. Antimycobacterial active agents

Infectious diseases are still one of the main human global health problems. Tuberculosis (TB) is one of the top ten human life threatening globally. It is the second cause of mortality after HIV/AIDS due to single infectious pathogen. Various pathogenic agents (*Mycobacterium* sp.) have been identified causing TB. Many drugs have been discovered for treating patients with TB but due to the side effects and drug resistant TB strains, novel therapeutical agents are still needed.^66^

**Fig. 15:**
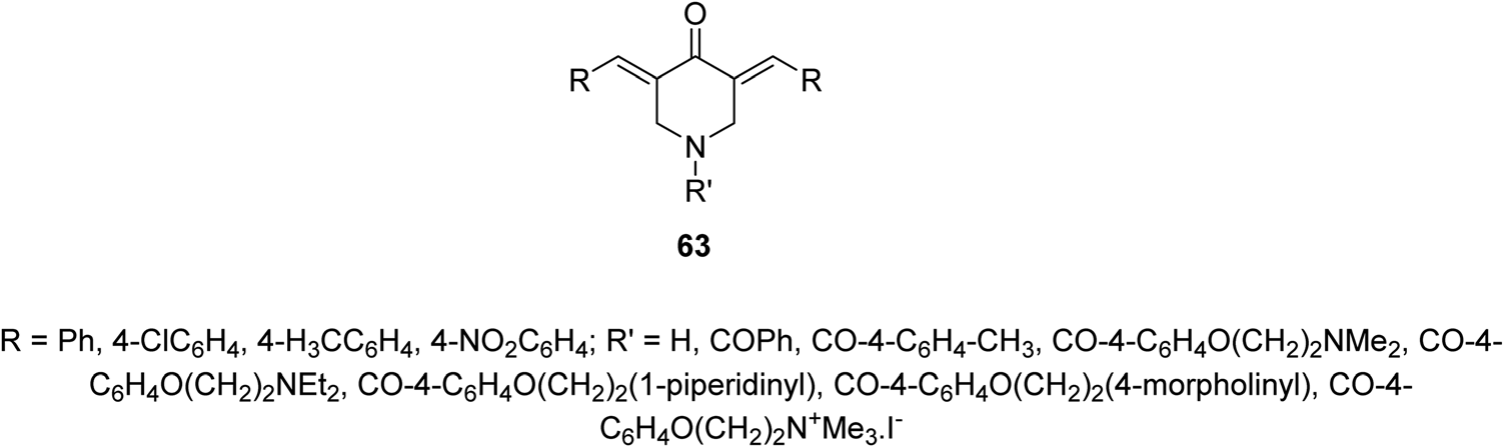
3,5-Bis(ylidene)-4-piperidones 63 with noticeable antimycobacterial properties.

A series of 3,5-bis(ylidene)-4-piperidones 63 were identified with noticeable antimycobacterial properties (*M. tuberculosis* H_37_Rv). Some of them revealed properties in the rat liver mitochondria respiration with swelling in mitochondria^67^ (Fig. 15).

**Scheme 29:**
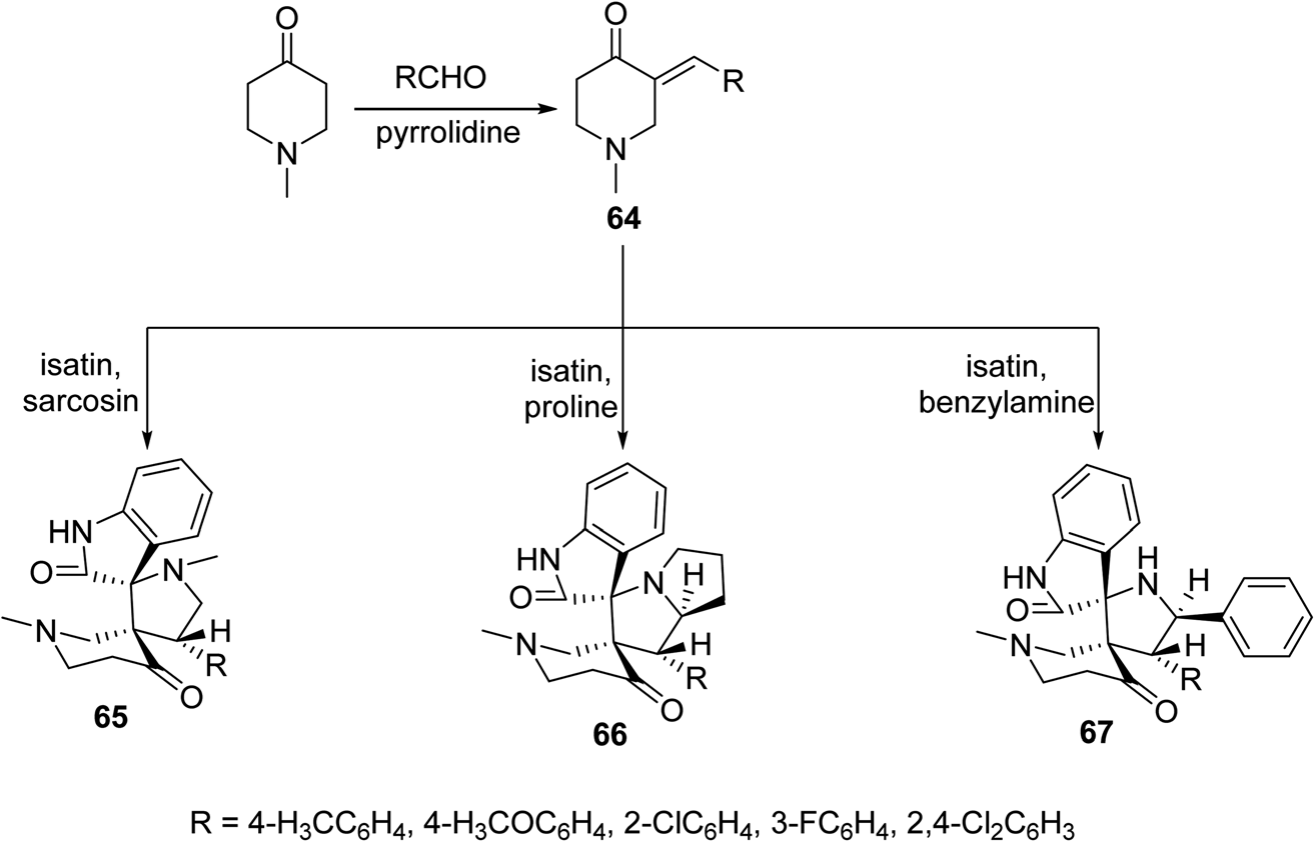
Spiro-heterocycles 65–67 of antimycobacterial properties.

Solvent-free microwave irradiation of 1-methyl-4-piperidone with aromatic aldehyde in presence of pyrrolidine afforded the corresponding 3-arylidene-4-piperidones 64. Azomethine ylide (derived from condensation of isatin with sarcosine, proline or benzylamine) cycloaddition to 64 in refluxing methanol yielded the corresponding spiro-heterocycles 65–67. Single crystal X-ray studies supported the synthesized structures. Antimycobacterial properties were observed by the synthesized spiro-heterocycles 65–67 against *M. tuberculosis*, multi-drug resistant *M. tuberculosis* and *M. smegmatis*^68^ (Scheme 29).

### 5.5. Antifungal active agents

**Scheme 30:**
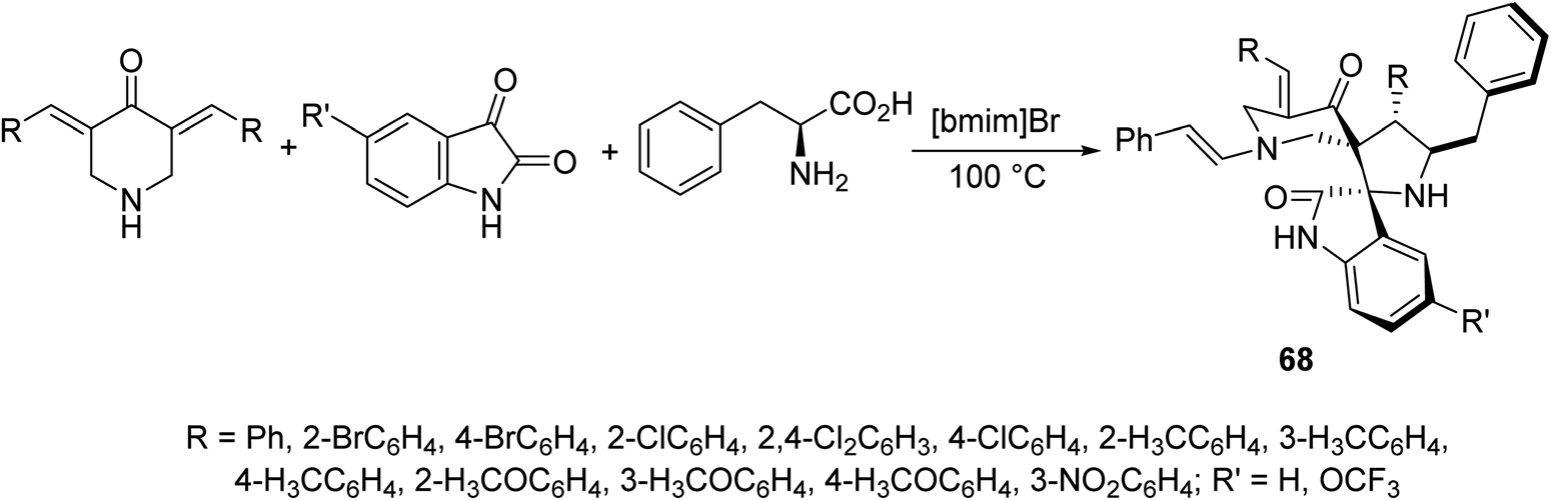
Dispiropyrrolidines 68 of antifungal properties.

Dispiropyrrolidines 68 were synthesized through multi-component domino azomethine ylide (formed from condensation of 2-amino-3-phenylpropanoic acid and isatin) dipolar cycloaddition reaction with the appropriate 3,5-bis(arylidene)-4-piperidones in ionic liquid “[bmim]Br” (Scheme 30). Antifungal properties of 68 were revealed against *Candida albicans* ATCC 10231. Some of the synthesized agents showed potent inhibitory properties relative to fluconazole (standard reference).^69^

### 5.6. Antimalarial active agents

Malaria is one of the most widely distributed infectious diseases in tropical and subtropical regions. *Plasmodium* sp. which is a protozoan organism transmitted to humans due to mosquito bites. Although many drugs are known to combat malaria emerging drug resistance means that new effective agents are still needed.^70^

**Fig. 16:**
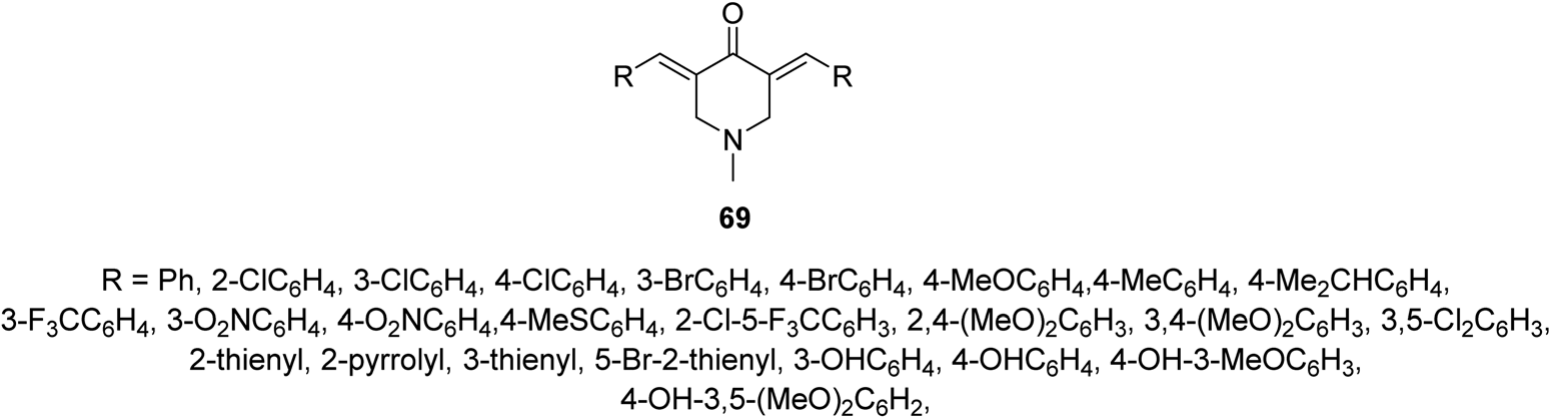
3,5-Bis(ylidene)(-*N*-methyl-4-piperidones) 69 of anti-plasmodial properties.

Anti-plasmodial properties were exhibited by 3,5-bis(ylidene)-*N*-methyl-4-piperidones 69 against chloroquine-sensitive Pf3D7, chloroquine-resistant PfINDO, and artemisinin-resistant PfMRA-1240 strains^71^ (Fig. 16).

**Scheme 31:**
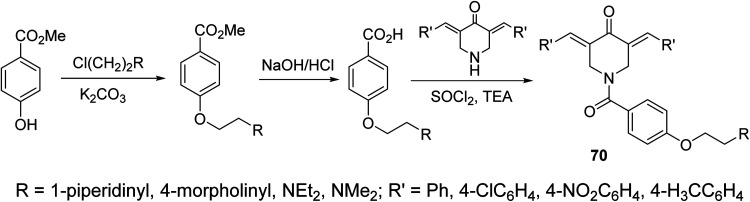
*N*-acyl-3,5-bis(ylidene)-4-piperidones 70 of antimalarial properties.

A series of *N*-acyl-3,5-bis(ylidene)-4-piperidones 70 were synthesized *via* dehydrohalogenation of the unsubstituted piperidone with the corresponding acid chloride (Scheme 31). Some of the synthesized agents revealed potent inhibitory properties against *Plasmodium falciparum* D6 and C235 (drug resistant) strains which also subjected to *Plasmodium berghei* revealing higher efficacy than chloroquine and mefloquine (standard reference drugs).^72^

### 5.7. Antiobesity active agents

**Scheme 32:**
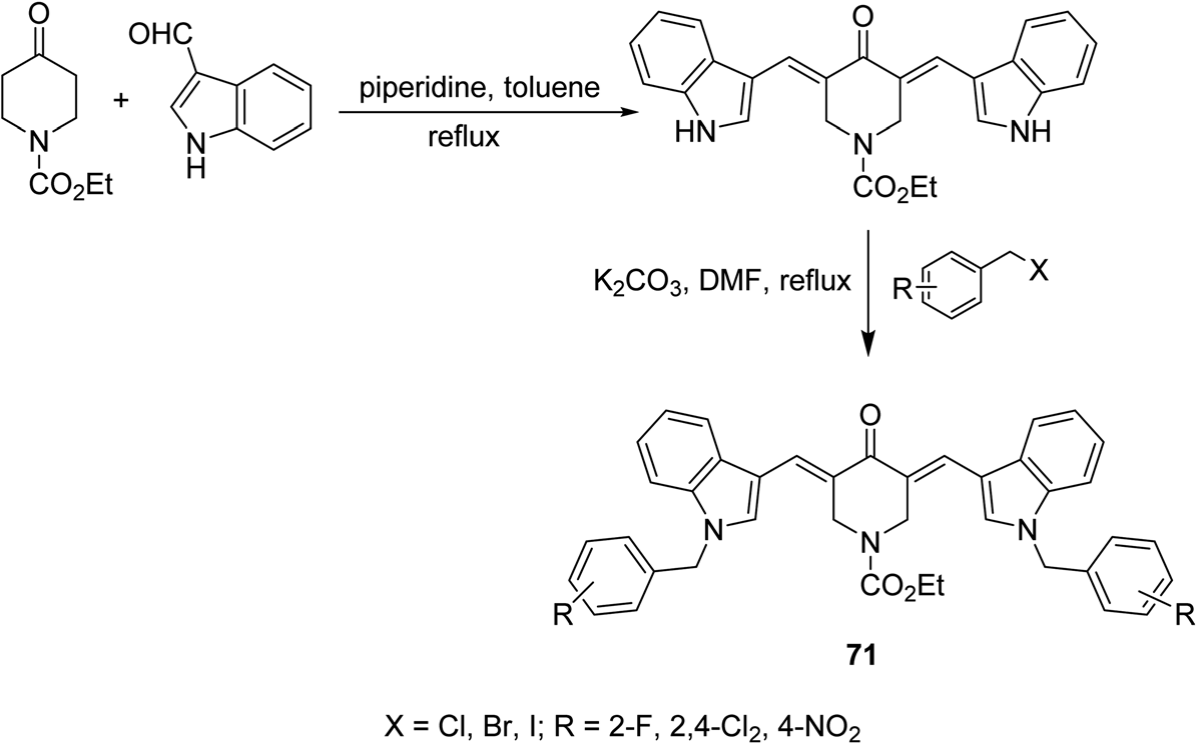
Synthetic route towards 1-ethoxycarbonyl-3,5-bis[(3′-indolyl)methylene]-4-piperidines 71 lipase inhibitory properties.

1-Ethoxycarbonyl-3,5-bis[(3′-indolyl)methylene]-4-piperidones 71 were synthesized through base catalyzed condensation (piperidine in refluxing toluene) of 1-ethoxycarbonyl-4-piperidine with 3-indolecarboxaldehyde followed by alkylation with various alkyl halides in refluxing DMF/K_2_CO_3_ (Scheme 32). Lipase inhibitory properties were revealed by the synthesized agents relative to orlistat (standard reference).^73^

## 6. Conclusion

Curcumin is an important natural compound with broad spectrum biological properties. Limitation of clinical application of curcumin is mainly due to its poor bio-availability *in vivo*. 3,5-Bis(ylidene)-4-piperidone scaffolds are considered a curcumin mimic with diverse promising bio-properties. The distinguished biological observations of curcumin mimics can be considered for optimizing high potent hits/leads accessible in drug discovery program. Many articles dealing with the biological properties of this scaffold have appeared. It has been also intensively utilized for construction of diverse potentially bioactive heterocycles.

## Conflicts of interest

There is no conflict to declare.

## Supplementary Material
